# Developmental Environment Effects on Sexual Selection in Male and Female *Drosophila melanogaster*

**DOI:** 10.1371/journal.pone.0154468

**Published:** 2016-05-11

**Authors:** Juliano Morimoto, Tommaso Pizzari, Stuart Wigby

**Affiliations:** Department of Zoology, Edward Grey Institute, University of Oxford, South Parks Road, Oxford OX1 3PS, United Kingdom; CNRS, FRANCE

## Abstract

The developmental environment can potentially alter the adult social environment and influence traits targeted by sexual selection such as body size. In this study, we manipulated larval density in male and female *Drosophila melanogaster*, which results in distinct adult size phenotypes–high (low) densities for small (large) adults–and measured sexual selection in experimental groups consisting of adult males and females from high, low, or a mixture of low and high larval densities. Overall, large adult females (those reared at low larval density) had more matings, more mates and produced more offspring than small females (those reared at high larval density). The number of offspring produced by females was positively associated with their number of mates (i.e. there was a positive female Bateman gradient) in social groups where female size was experimentally varied, likely due to the covariance between female productivity and mating rate. For males, we found evidence that the larval environment affected the relative importance of sexual selection via mate number (Bateman gradients), mate productivity, paternity share, and their covariances. Mate number and mate productivity were significantly reduced for small males in social environments where males were of mixed sizes, versus social environments where all males were small, suggesting that social heterogeneity altered selection on this subset of males. Males are commonly assumed to benefit from mating with large females, but in contrast to expectations we found that in groups where both the male and female size varied, males did not gain more offspring per mating with large females. Collectively, our results indicate sex-specific effects of the developmental environment on the operation of sexual selection, via both the phenotype of individuals, and the phenotype of their competitors and mates.

## Introduction

Sexual selection favours traits that confer an advantage in intra-sexual competition in both sexes [1]. While historically, sexual selection studies have focused on males, it is now appreciated that intra-sexual competition can also play an important role in the evolution of females, which in turn affect males responses to female adaptations [2, 3]. It is therefore important that studies on sexual selection consider both sexes, because resolving the evolution of sex roles and the nature of sexual conflict hinges largely on understanding the mechanisms that cause sex-specific patterns of sexual selection.

Traditionally, intra-sexual competition was considered exclusively over mating opportunities, and the strength of sexual selection has been measured by the slope of the linear univariate regression of offspring number against number of mates—the “Bateman gradient” [4–7]. Hence, the Bateman gradient explicitly captures only one component of pre-copulatory sexual selection: the number of mates (i.e. “mating success”). By showing that male Bateman gradients are often steeper than female gradients, this approach has been instrumental in defining sex roles [8–10]. Yet, it is becoming increasingly evident that other factors can influence the relationship between mate number and offspring number, particularly for males, where variation in paternity share due to post-copulatory sexual selection and variation in female productivity can be important (e.g. [11–13]). This indicates that the total number of offspring sired by a male (i.e. his “reproductive success”) is best described through a multivariate approach as follows:

T=M*P*N+ε Eq 1

where *T* is the total number of offspring produced, *M* is the number of females mated by a male, *P* is his average paternity share of the offspring produced by his mates, *N* is the average number of eggs produced by his mates and ε is an error term with 0 mean [11, 14]. In this case, the multivariate model incorporates measures of both pre- and post-copulatory sexual selection and allows the investigation of their relative contribution to the total number of offspring sired by males [11]. Therefore, the multivariate approach substantially deepens our understanding of the factors that determine variation in the number of offspring sired by different males [11, 12, 15].

The interpretation of the female Bateman gradient has also attracted considerable debate [12, 16–18]. For instance, it is becoming increasingly clear that female Bateman gradients can be steeper than originally assumed (e.g. [2, 3, 12, 19–22]). However, the causality of this relationship is not always clear. In principle, a positive female Bateman gradient can measure sexual selection on female mate number, for example when mating provides cumulative direct benefits to females [17, 23]. However, positive female Bateman gradients can also arise as a result of non-causal or inverse associations between mate number and the number of offspring produced [16, 18, 24], for example when inherently more fecund females either attract or require more mates [12, 22].

A likely modulator of sex-specific patterns of sexual selection is the pool of resources available to individuals to allocate to traits [25] (i.e. individual’s environmental conditions) and the pool of resources available for individual’s competitors (i.e. social condition). Both the individual’s environmental and social conditions can influence the strength of sexual selection, for example through the modulation of adult competitive ability, mate preferences, or productivity and body size, if body size is correlated with environmental conditions that affect any of these traits [26, 27]. For instance, Janicke, David [28] recently showed that fluctuations in adult food availability levels can affect body weight, reproductive traits and the strength of sexual selection on a simultaneously hermaphroditic snail, *Physa acuta*. In food-restricted snails, mating was not significantly associated with increments in offspring number, resulting in non-significant Bateman gradients for both male and female roles, in contrast to larger, food-unrestricted snails where gradients were significantly positive. Adult diet manipulation rather than developmental diet implies relatively rapid plastic responses. However, the study of simultaneous hermaphrodites makes it difficult to disentangle the independent effect that diet may have on male and female roles from its influence on trade-offs in sex allocation within individuals.

In insects, including *Drosophila melanogaster*, adult body size is often mediated by the environment during development [29–32], which in turn tends to positively correlate with female productivity (i.e. large females produce more eggs than small females) and male quality [26]. Adult body size is expected to be under productivity selection (in females) and sexual selection (in males) in adult insects [3, 26, 33–35]. A recent study showed that males with small body size (raised at high larval density) have reduced reserves of seminal fluid but invest proportionally more of this seminal fluid *per* mating than large males (raised at low larval density)[36]. Given that seminal fluid is limited in supply in *D*. *melanogaster* [37–39] small males may have a reduced ability to transfer multiple full-sized ejaculates. If so, being small could potentially reduce the benefits of multiple matings for males–in which case we would expect to observe reduced Bateman gradients–and could also modulate post-copulatory competitiveness [40]. Furthermore, both large and small males invest more seminal fluid when mating with large females, suggesting that males can adjust their ejaculate investment depending on the body size of their mates [36]. Importantly, the developmental environment tends to affect male fitness more than female fitness [41], suggesting that environmental conditions can have sex-specific direct (i.e. on the individual) or indirect (i.e. on the individual’s mate) effects on selective forces. It is consequently reasonable to expect that there may be links between variation in the developmental environment, adult body size and the strength and form of sexual selection within adult populations.

Together, these previous studies indicate that environmental effects, particularly larval environment-mediated effects on adult body size or on traits associated with body size, have the potential to influence the strength of both pre- and post-copulatory sexual selection within populations. However, despite the growing interest in ecological factors affecting reproduction [28, 29, 41–44], the effects of the developmental environment on patterns of sexual selection have received little attention. In this study, we manipulated adult traits, including body size, by varying larval density in *D*. *melanogaster*. Increasing larval density limits the quantity and quality of the food available per larvae, results in reduced adult body size, and has far-reaching consequences for male and female reproduction [29, 30, 45–49]. Larval density may also signal as an index of population density, which is expected to play a central role life-history traits in species with high reproductive rates such as *D*. *melanogaster* and other insects [50], and therefore has important ecological and evolutionary implications. For example, high developmental densities might provide cues of intense intrasexual competition in adulthood [51, 52]. Here, we tested how larval density influences reproductive behaviour and the strength of sexual selection, as measured by the Bateman gradient. Although, as described above, a manipulation of larval density is likely to affect multiple traits and impose different selective pressure on the larvae [29, 45, 53], for conciseness we refer to the set of experiments and groups according to the body size of adults, because this is the most striking adult phenotype from the larval density manipulation and is consistent with terminology in previous literature (e.g. [29, 43]).

In principle, the developmental environment can influence the fitness of a focal individual directly by influencing a focal individual’s own phenotypes, and by modulating the phenotypes of other group members (i.e. the competitors and potential mates of the focal individual), which can in turn feedback on the fitness of the focal individual. We used an experimental approach to explore these focal and group effects within each sex, by assembling groups of adults in which larval-density manipulations had resulted in body sizes which were constantly large, constantly small or varied in either sex or simultaneously in both sexes. Firstly, we investigated how the larval density manipulations on individuals and on group composition influenced the number of mates, mating frequency and the number of offspring produced by males and females. Then, we investigated how the larval density manipulations on individuals and on group composition affected the strength of sexual selection in females and males.

## Predictions

### Reproduction

- Overall, based on previous literature, we expected large individuals to mate more frequently, obtain more mates and produce more offspring than small individuals

### Sexual selection

- We expected female Bateman gradients to be steeper in female mixed size social environments due to the association between body size, number of mates and offspring production. Because this effect relies solely on female’s physiological and behavioural traits, we did not predict effects of male body size variation in this pattern.
- We expected male Bateman gradients to be generally positive regardless of male body size. However, we also expected the social environment to modulate the strength of sexual selection on male size both before and after copulation. For instance, we predicted stronger post-copulatory sexual selection on large males in homogenous social environments (where competitors are similarly large and equally good competitors) than in heterogeneous social environments (where large males may outcompete small males). Strong post-copulatory sexual selection may increase ejaculate investment and reduce the benefits of multiple copulations. As a result, we also expected the Bateman gradient of large males to be reduced for large when experiencing homogeneous social environments.
- Finally, when manipulating both sexes body size, we expected to strengthen sexual selection on male size by enabling large males to outcompete small males over large, more fecund females and their eggs, and by enabling large females to outcompete small females over access to large males and their sperm.

## Material and Methods

### Fly stocks and culture

We used a wild-type stock of *D*. *melanogaster* that was collected in Dahomey (Benin) in North Africa in 1970 and has been maintained in large (>5,000 individuals) outbred populations in cages with overlapping generations [54]. Focal males were wild-type Dahomey, while competitor males and experimental females carried the recessive *sparkling*^*poliert*^ mutation (*spa*), which had been backcrossed into the Dahomey genetic background for more than 5 generations. The *spa* mutation produces a rough-looking eye phenotype when homozygous [55] and is commonly used in sperm competition assays to assign paternity (Fricke, Martin [56]). All fly stocks were maintained, and all experiments conducted, at 25°C on a 12:12 light:dark cycle in a non-humidified room and were fed with standard sugar-yeast-maize-molasses medium with excess live yeast granules.

### Larval density manipulation to vary adult body size

Following the protocol of Clancy and Kennington [57], we collected eggs from population cages and pipetted eggs. We used the following densities to manipulate the developmental environment: high density ~100 larvae/mL of food (~400 larvae in a 34ml vial containing ~4mL fly food), and low density ~ 4 larvae/mL of food (~200 larvae in a 170ml bottle containing ~50mL fly food), which generated adult flies of small and large body size, respectively. Using this protocol we previously obtained adult females and males of significantly different and non-overlapping body size classes, and with comparable distribution of the variance in the body size (mean mass [mg] ± SE: females, large = 1.60 ± 0.06, small = 0.814 ± 0.08, F_1,30_ = 272.1; males, large = 0.87 ± 0.03, small = 0.60 ± 0.06, F_1,30_ = 85.7; p < 0.0001 for all within-sex comparisons (from Wigby, Perry [36]). Using a larger container for the low-density manipulation allowed us to keep the overall population size per container of a similar order of magnitude across the different larval manipulation regimes (i.e. all flies were grown in a container with hundreds of conspecifics) (see [40]). We subsequently refer to the body sizes of adult flies as shorthand for their larval density manipulation: i.e. “large” or “small” to indicate those individuals grown in low and high larval density, respectively. Virgin flies were collected within 8 hours of eclosion and kept in vials of same-sex and same-larval manipulation groups of 15–20 individuals for 2–5 days prior to experiments. In order to track individual flies throughout the behavioural observations we marked all flies of both sexes using 4 colours of acrylic paint—white, yellow, red and orange–one colour *per* individual *per* sex. 24h before experiments began, female flies were randomly allocated to one of these 4 paint colours and were marked on the thorax [58, 59]; this was done for females across all treatments. Focal males were painted in half of the vials with white colour and with yellow colour in the remaining half of the vials. The three competitor males were assigned the remaining paint colours. After paint marking, flies were allocated to their experimental groups (see below), and held in single-sex vials prior to the start of experimentation.

### Experimental design

**Fig 1:**
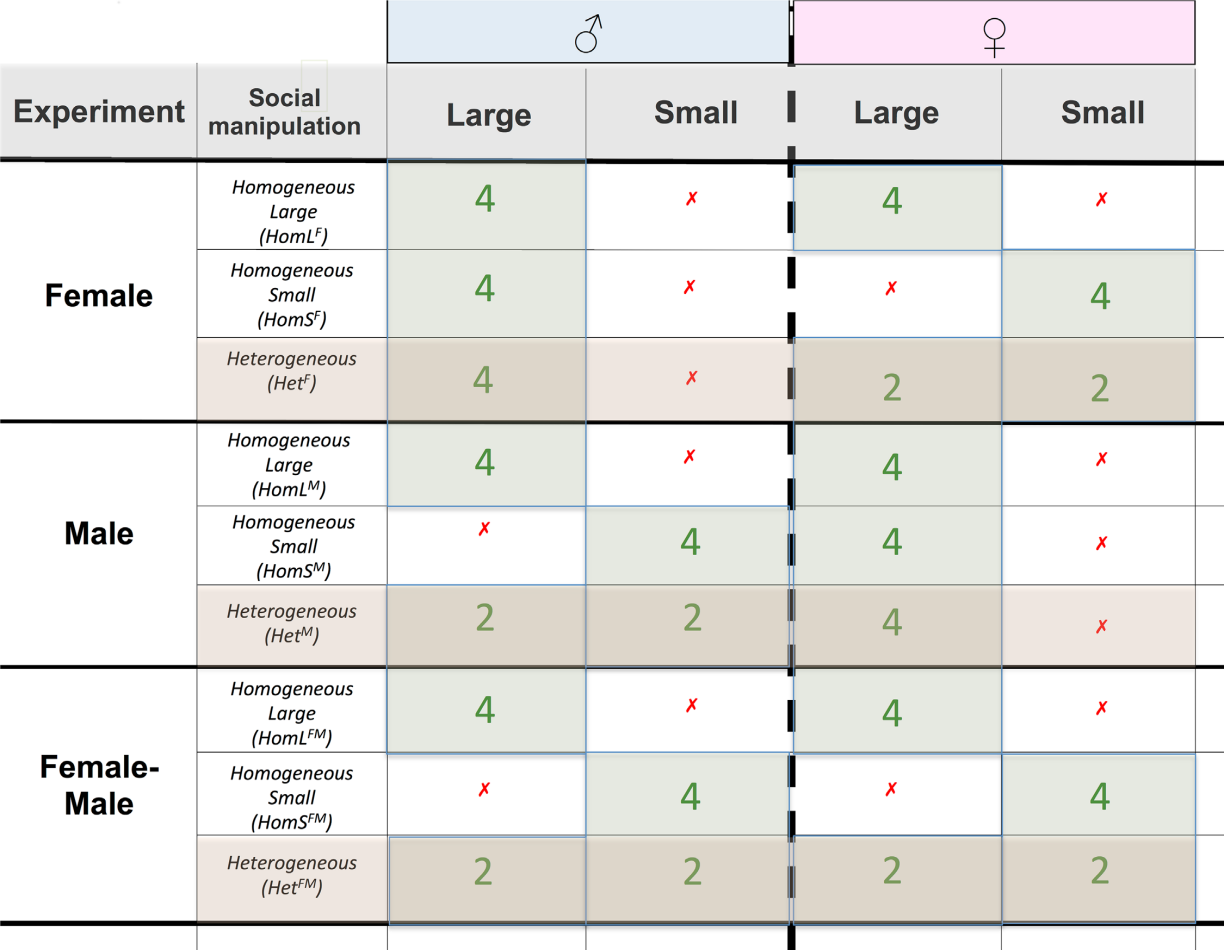
Diagram of the experimental design. We investigated the effects of the developmental environment on the strength of sexual selection. Because larval density strongly influences adult body size we refer to low-larval density flies as “large” and high larval density flies as “small”. *The Female Experiment*–experiment varying female larval density, keeping males constant (low larval density). Homogeneous social environments consisted of 4 large or 4 small females in addition to 4 large males. Heterogeneous social environments consisted of 2 large *and* 2 small females in addition to 4 large males. *The Male Experiment*–experiment varying male larval density, keeping females constant. Homogeneous social environments consisted of 4 large or 4 small males in addition to 4 large females. Heterogeneous social environments consisted of 2 large *and* 2 small males. *The Female -Male Experiment*–experiment varying both male and female larval density. Homogeneous social environments consisted of 4 large males and females or 4 small males and females. Heterogeneous social environments consisted of 2 large males and females *and* 2 small males and females. In all experiments one male, out of each group of four, was the focal individual for which we obtained paternity data (see methods).

Our experimental design for measuring the strength of sexual selection in groups of flies was based on that of Bjork and Pitnick [60]. Briefly, replicate vials contained 4 flies of each sex (i.e. 8 flies per vial) were allowed to interact for 4 hours per day over 4 days, before being discarded. Within each vial, three of the males and all females were *spa*, and one male–the “focal” male–was wild-type, which allowed us to assign the paternity to the focal male (because *spa* is recessive). We conducted three experiments to manipulate social environments. We varied: 1) female adult body size, while keeping male size constant (the “Female Experiment”); 2) male adult body size, while keeping female size constant (the “Male Experiment”); and 3) both male and female body size simultaneously (the “Female-Male Experiment”, see Fig 1).

The Female Experiment*–*In this experiment, female body size was manipulated while male body size was kept constant (i.e. large body size). The heterogeneous social environment (Het^F^) comprised of two large (HetL^F^) and two small (HetS^L^) females with the four large males. The homogeneous social environment groups consisted of females with constant body size class (i.e. all from the same larval density rearing environments)–i.e. either all large (HomL^F^) or all small (HomS^F^) females.

The Male Experiment*–*In this experiment, male body size was manipulated while female body size was kept constant (i.e. large body size). The heterogeneous social environment (Het^M^) comprised of two large (HetL^M^) and two small (HetS^M^) males with the four large females. For the heterogeneous social environments, focal males had small body size in half of the replicates (N = 10) and large body size in the other half (N = 10) of the replicates. The homogeneous social environment groups consisted of males with constant body size class (i.e. all from the same larval density rearing environments)–i.e. either all large (HomL^M^) or all small (HomS^M^) males.

The Female-Male Experiment*–*In this experiment, both male and female body size was manipulated. The heterogeneous social environment (Het^FM^) comprised of two large males and two large females (HetL^FM^) and two small males and two small females (HetS^FM^). For the heterogeneous social environments, focal males had small body size in half of the replicates (N = 10) and large body size in the other half (N = 10) of the replicates. The homogeneous social environment groups consisted of males and females with constant body size class (i.e. all from the same larval density rearing environments)–i.e. either all large (HomL^M^) or all small (HomS^M^) individuals.

Note that HomL^F^, HomL^M^, and HomL^FM^ are identical treatments across each experiment, and the label differences simply represent different experiments they were part of (i.e. Female Experiment, Male Experiment, and Male-Female Experiment, respectively).

Therefore, throughout our experiments, individuals in the homogeneous environments were exposed to individuals with the same body size of the sex being manipulated, whereas individuals in the heterogeneous environments were exposed to both small and large individuals. We made 20 replicate vials for each heterogeneous social environment (see below), and 10 for each homogeneous social environment group (i.e. 40 vials for each experiment, 120 in total).

We recorded all matings during the 4-hour interaction periods on each of the 4 days of the experiment. Thus, we scored every mating for every individual, allowing us to calculate both the mating frequency and number of mates for each individual. Between interaction periods females were separated from males and placed individually in fresh oviposition vials, and were allowed to lay eggs for twenty hours (i.e. until the interaction period the following day). Flies were discarded in the fifth day. Oviposition vials were retained and all emerging adults were counted after eclosion (13–15 days after the day of oviposition to ensure all flies had sufficient development time) to measure the number of offspring produced by females. Because all females and the three competitor males carried the recessive *spa* mutation, we could assess the number of offspring sired by focal males: by counting how many offspring were *spa* and how many were wild-type we could calculate the focal (wild-type) male’s paternity share. Paternity share was calculated as the sum of wild-type offspring produced by all the mates of the focal male divided by the total offspring (*spa* plus wild-type) produced by those females. Because the three competitor males were all *spa*, we did not have individual paternity data for these males–thus, only the competitor male’s paternity share could be measured. We also calculated “*per mating”* female offspring production as the total number of offspring produced by a female divided by the total number times that female mated. We used female *per mating* offspring production as an estimate of the expected average reproductive value of each additional mating for males (Note that offspring *per* mating is not equivalent to the Bateman gradient, which measures the slope of the observed relationship between number of *mates* and number of offspring–see below). Final sample sizes were as follows: The Female Experiment, n = 156 females (HomL^F^ = 40, HomS^F^ = 40, Het^F^ = 76), n = 40 focal males (HomL^F^ = 10, HomS^F^ = 10, Het^F^ = 20); The Male Experiment, n = 149 females (HomL^M^ = 37, HomS^M^ = 38, Het^M^ = 74), n = 40 males (HomL^M^ = 10, HomS^M^ = 10 and Het^M^ = 20); The Female-Male Experiment, n = 155 females (HomL^FM^ = 39, HomS^FM^ = 39, Het^FM^ = 77), n = 40 males (HomL^FM^ = 10, HomS^FM^ = 10, Het^FM^ = 20). In some vials a single focal female (but no males) died during the experiment: the Female Experiment, Het^F^ group (4 vials); Male Experiment, HomL^M^ (3 vials), HomS^M^ (2 vials) and Het^M^ (6 vials); Female-Male Experiment, HomL^FM^ (1 vial), HomS^FM^ (1 vial) and Het^FM^ (3 vials). Males that failed to mate were kept in the analysis because failing to mate is likely a consequence of sexual selection. There were no non-mating females.

### Data analysis

The three experiments were conducted independently so we carried out analyses separately for each experiment. Because we had complete paternity data for one focal male per replicate group (see methods above), whereas we had data for every female in each replicate group, the analyses were also conducted separately for each sex. Therefore, for males, we used the single focal individual per vial as the unit of replication, whereas for females we analysed data from all individuals but included the vial as a covariate in all models, to account for pseudoreplication. We first characterised the effects of developmental environment on female and male reproductive traits, and subsequently measured the effects on sexual selection.

#### Male and female reproductive trait analyses

First we investigated the potential effects of female deaths (“*DF”*, dead females) on the results. We pooled the data of our three experiments and created a binary variable *DF*, which contained information on the occurrence (value = 1) or not (value = 0) of a female death in the social environment of a particular individual. A significant effect of *DF* means that the death of an experimental female increased or decreased reproduction of the remaining individuals in that social group. We used quasipoisson GLMs, which account for overdispersion of the data, to evaluate whether *DF* had a significant effect on the number of mates, mating frequency and number of offspring of both females and focal males. There was a significant effect of *DF* on focal male estimates of number of mates and offspring (see ‘S1 Text’) and, therefore, *DF* was retained in all analyses of focal male reproduction and sexual selection. Then, we tested for the effect of paint marking, but found no effect of colours on either females or focal male reproduction (see ‘S1 Text’); we therefore excluded colours from the final analysis.

We then focused on both females and focal male reproduction. We used generalised linear models (GLM) (family = ‘quasipoisson’) to test for differences between levels (see below) in male and female mating frequencies and mate numbers, in the number of offspring produced by females, and in female *per mating* offspring (the number of offspring produced by a female divided by the number of times that female mated). We used a ‘quasibinomial’ GLM to investigate the average proportion of offspring sired by a focal male. In our models, the explanatory variables were social environment (“social”, which was either homogenous or heterogeneous: see experimental design above), the body size of the sex under consideration, and the interaction between social environment group and body size (i.e. social*body size) whenever the sex under consideration varied in body size (i.e. females in the Female Experiment, males in the Male Experiment, and both sexes in the Female-Male Experiment). If the sex being analysed did not vary in body size (i.e. males in the Female Experiment, females in the Male Experiment), we used GLMs with “social” environment treated as the variable with three levels (e.g. Hom^L^, Hom^S^ and Het). For models in which “social” was the explanatory variable with three levels and the analysis showed p-value ≤ 0.05, we performed a post-hoc “SNK-test” to investigate the difference between the means of the three levels [61]. P-values are based on F-statistics for all GLM models.

#### Sexual selection analyses

For females, we measured the strength of sexual selection on mate number, as β, the slope of a linear regression of standardised offspring number (*T*) on standardised number of mates (*M*):

T(M)=(β*M)+ε Eq 2

We standardised *T* by dividing the offspring number of each individual by the mean number of offspring produced by all members of that sex in the group (to give relative reproductive success; see Arnold 1994). Similarly, we standardised *M* as the following: we subtracted the mean number of mates of all individuals of that sex in the group from the number of mates of each individual, and divided the resultant value by the standard deviation of the number of mates of all individuals of that sex in the group. In doing so, we scaled *M* to have a mean of zero and a standard deviation of unity and *T* to have a mean of 1 (Arnold 1994). We also investigated quadratic effects (see S1 Text) on female Bateman gradients [12] because *T* might peak at a given number of males mated followed by a plateau or decline (Jones 2009). Whenever the quadratic term was non-significant, it was removed from the analysis.

For males we first characterised sexual selection in a qualitative way, by decomposing variance in *T* as follows:

$$Var(M\;*\;N\;*\;P)\;=\;{\bar{N}}^{2}\;{\bar{P}}^{2}\;Var(M)+\;{\bar{M}}^{2}{\bar{P}}^{2}\;Var(N)+\;{\bar{N}}^{2}\;{\bar{M}}^{2}\;Var(P)+\;2\;\bar{M}\;\bar{N}\;{\bar{P}}^{2}\;Cov(M,\;N)+\;2\;\bar{M}\;\bar{P}\;{\bar{N}}^{2}\;Cov(M,\;P)+\;2\;\bar{P}\;\bar{N}\;{\bar{M}}^{2}\;Cov(P,\;N)\;+D$$ Eq 3

where $\bar{M}\bar{,\;P}$, and $\bar{N}$ are the mean values of *M*, *P* (average paternity share) and *N* (average number of adult offspring produced by a male’s mates), and D includes the variance in the error term **ε** [11, 15, 62]. This approach enables us to measure the relative contributions of *M*, *P* and *N* and their covariances to *T* (see [11, 28]). We also used commonality analysis (CA) [63], which decomposes the explained variance of the multivariate linear model (see below) into percentage of variance explained by one (i.e. *unique* explained variance) or a set of variables (i.e. *common* explained variance) [64, 65], to explore the relative contributions of *M*, *P*, *N*, and their covariate to variation in the number of offspring sired by males (see S1 Text).

Although the decomposition of variances is useful to make qualitative comparisons across social treatments, the quantitative interpretation of variances and covariances can be difficult [66]. To overcome this limitation, we measured linear selection gradients on male *M*, *P* and *N* using a multivariate model based on Eq 1, which was composed of *T* (dependent variable), *M*, *P* and *N* (independent variables) as well as “total vial productivity” (*VP*), which accounted for differences in within-vial offspring productivity, and *DF*, which accounted for female deaths in the social environment (see above). The model was (in ‘R’ notation):

st(T)∼st(M)+st(P)+st(N)+VP+DF Eq 4

where *st()* indicates the standardised values of the variables *T*, *M*, *P* and *N* (see above). This multivariate approach allowed us to investigate the effects of the larval manipulation on the Bateman gradient (i.e. gradient of *T* regressed over *M*, controlling for all covariates), the paternity share gradient (i.e. gradient of *T* regressed over *P*, controlling for all covariates) and the mate productivity gradient (i.e. *T* regressed over *N*, controlling for all covariates). We standardised *P* and *N* in the multivariate model dividing *P* and *N* of each individual focal male by the mean number of *P* and *N* of all focal males in the group, respectively.

For both males and females we first tested whether the Bateman gradients of the homogenous and heterogeneous groups significantly differed from 0 in each experiment. We also analysed the gradients of small and large flies within heterogeneous groups (e.g. Het^F^, Het^M^, Het^FM^) separately. This allowed us to determine whether selection was acting differently on small *versus* large flies within heterogeneous groups. For males we additionally tested paternity and mate productivity gradients, using the same approach as for Bateman gradients above (i.e. for both homogenous and heterogeneous groups, and for small and large males separately in heterogeneous groups). Finally, we tested the influence of the social environment by comparing the selection gradients on small and large individuals in homogenous versus heterogeneous groups. To do this we fitted a multivariate linear model for large and small individuals separately that included interaction terms of *M*, *P* and *N* and social environment composition (*SEC*) (i.e. M*SEC, P*SEC and N*SEC), in addition to our covariates of *VP* and *DF*. We present p-values from *t-*statistics for all multivariate linear models. All analyses were performed using the R software (version 3.0.2, [67]).

## Results

### Patterns of female reproduction

**Fig 2:**
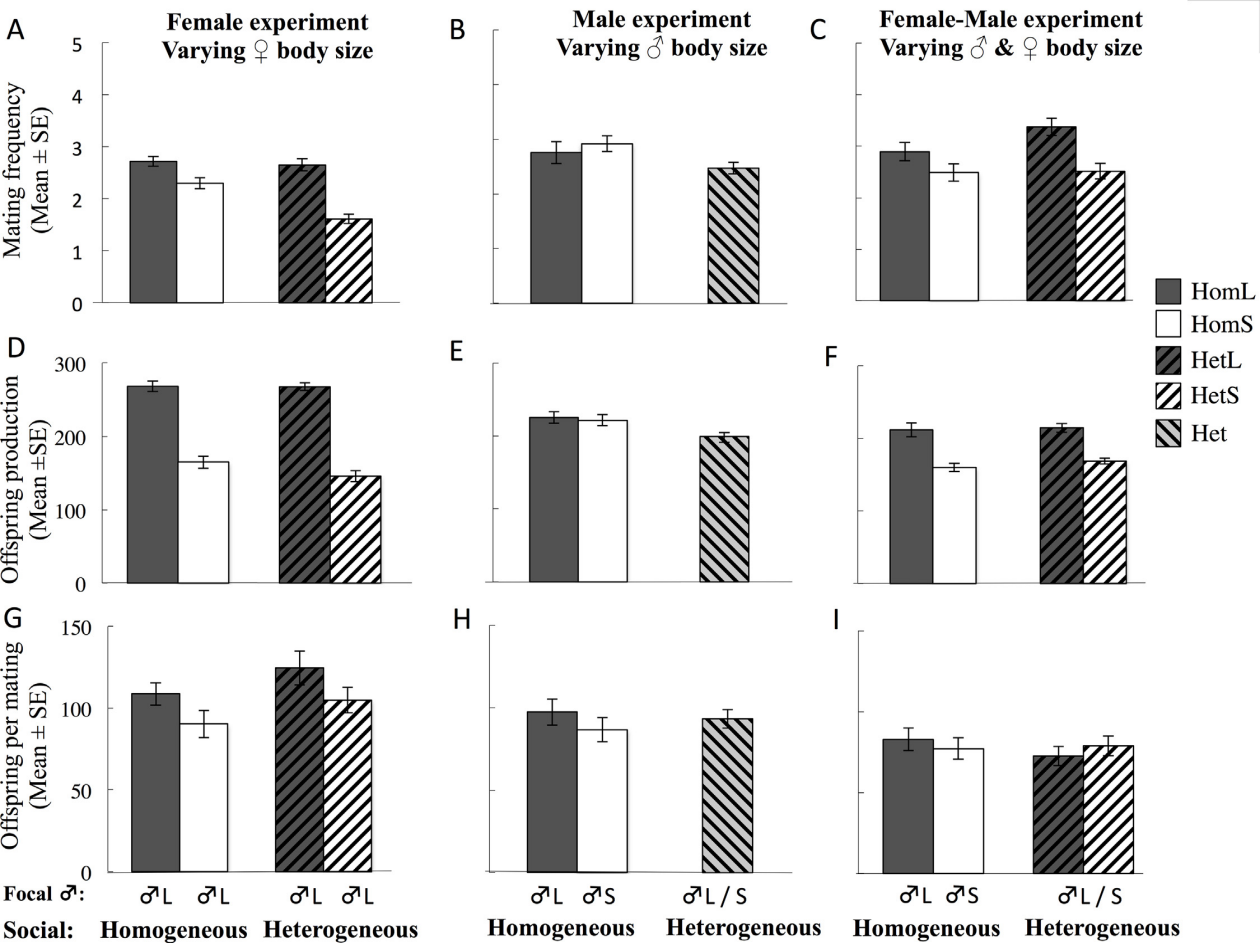
Female developmental environment and adult reproduction. (a–c) Female mating frequency: (a) Female Experiment; (b) Male Experiment and (c) Female-Male experiments. (d–f) Female offspring production: (d) Female Experiment; (e) Male Experiment and (f) Female-Male experiments. (g-i) *per mating* female offspring production: (g) Female Experiment; (h) Male Experiment and (i) Female-Male experiments. Solid dark grey–Homogeneous Large, Solid white–Homogeneous Small, Dark grey striped from bottom left to upper right–Heterogeneous Large, White striped from bottom left to upper right–Heterogeneous Small, Light grey striped from bottom right to upper left–Heterogeneous (combined Large and Small).

Large adult females (those reared at low larval density) mated significantly more frequently, mated with significantly more mates, and produced significantly more offspring, than small females (those reared at high larval density) across all experiments in which female body size was manipulated (i.e. Female Experiment and Female-Male experiment) (Table 1, Fig 2A–2F, S1 Fig). Large females had higher offspring *per mating* in the Female Experiment (i.e. where female body sized varied), but this difference was absent in the Female-Male Experiment (where both sexes varied in body size; Table 1, Fig 2G–2I). Social environment (i.e. homogenous or heterogeneous) affected female mating behaviour in the Female Experiment, where females in the homogeneous social environment mated significantly more frequently, and with more mates, than females in heterogeneous environment (Table 1, Fig 2A, S1 Fig). The social environment also affected female offspring production in the Male Experiment, where females in the heterogeneous male size environment (i.e. exposed to large and small males) produced significantly fewer offspring than females in a homogeneous male social environment (i.e where all males were had large body size; Table 1, Fig 2B, post-hoc SNK test HomL > HomS > Het (**α** = 0.05))). We also found a significant interaction between social environment and female body size (i.e. social*body size) on female mating frequency in the Female Experiment, which was driven by a stronger reduction in mating frequency for small females in heterogeneous social environments relative to homogeneous social environments (Table 1, Fig 2A). We did not detect an effect of social environment or an interaction between social environment and body size on offspring production or offspring per mating of females in the Female Experiment, or on mating frequency, number of mates, or offspring per mating in the Male Experiment, or on any of the reproductive measures in the Female-Male Experiment (Table 1). The exclusion of a single female in the Female Experiment that mated but did not produce offspring does not qualitatively change the results except for total offspring production, in which the interaction term social * body size, that was marginally non-significant (p = 0.081), becomes significant (p = 0.024). This result would indicate that the reduction in offspring production in the heterogeneous groups was stronger for small than for large females.

**Table 1:** Development effects on reproduction. Analysis of focal male and female mating frequency, number of mates, absolute offspring production, proportion of focal male’s offspring production and female *per-mating* offspring production.

Response variable	Factor	Female experiment				Male experiment				Female-Male experiment			
		Varying ♀ body size				Varying ♂ body size				Varying ♂ &♀ body size			
		♂		♀		♂		♀		♂		♀	
		F-value	p-value	F-value	p-value	F-value	p-value	F-value	p-value	F-value	p-value	F-value	p-value
	Social	0.384	0.683	5.157	**0.024**	0.094	0.760	2.838	0.061	0.485	0.490	2.537	0.113
Mating frequency	Size	-	-	21.551	**<0.001**	0.377	0.542	-	-	0.019	0.890	14.175	**<0.001**
	Vial	-	-	1.087	0.298	-	-	1.178	0.279	-	-	0.079	0.779
	DF	0.568	0.455	-	-	0.141	0.708	-	-	0.819	0.371	-	-
	Social*Size	-	-	5.687	**0.018**	8.373	**0.006**	-	-	0.832	0.367	1.394	0.239
	Social	1.105	0.342	6.616	**0.011**	2.994	0.092	0.392	0.676	0.419	0.521	0.216	0.642
Number of mates	Size	-	-	9.929	**0.001**	0.478	0.493	-	-	0.046	0.830	9.948	**0.001**
	Vial	-	-	4.924	**0.027**	-	-	2.121	0.147	-	-	0.081	0.775
	DF	0.070	0.792	-	-	0.033	0.854	-	-	10.884	**0.002**	-	-
	Social*Size	-	-	0.885	0.348	1.171	0.286	-	-	0.205	0.653	0.255	0.614
	Social	3.356	**0.046**	0.882	0.349	0.132	0.717	4.571	0.011	4.622	**0.038**	0.995	0.320
Offspring production	Size	-	-	268.830	**<0.001**	0.324	0.572	-	-	2.459	0.125	52.286	**<0.001**
	Vial	-	-	1.531	0.217	-	-	0.073	0.786	-	-	1.315	0.253
	DF	1.547	0.221	-	-	0.019	0.889	-	-	6.065	**0.018**	-	-
	Social*Size	-	-	3.081	0.081	4.883	**0.033**	-	-	0.766	0.387	0.281	0.596
	Social	1.533	0.230	-	-	0.060	0.807	-	-	0.626	0.434	-	-
Proportion of focal	Size	-	-	-	-	10.812	**0.002**	-	-	0.385	0.538	-	-
male's offspring	DF	1.496	0.229	-	-	0.000	0.982	-	-	0.049	0.825	-	-
	Social*Size	-	-	-	-	5.296	**0.027**	-		0.070	0.792	-	-
	Social	-	-	3.443	0.065	-	-	0.482	0.618	-	-	0.480	0.489
Offspring	Size	-	-	5.079	**0.025**	-	-	-	-	-	-	0.002	0.764
*per* mating	Vial	-	-	0.249	0.618	-	-	0.318	0.573	-	-	0.010	0.918
	Social*Size	-	-	0.006	0.936	-	-	-	-	-	-	0.872	0.351

F-values and p-values are given. “Social” refers to the composition of the social environment (i.e. homogeneous or heterogeneous) (see Methods). The Female Experiment–varying female body size; The Male Experiment–varying male body size; The Female-Male Experiment–varying both male and female body size. Bold–p–value ≤ 0.05.

### Sexual selection on females

**Fig 3:**
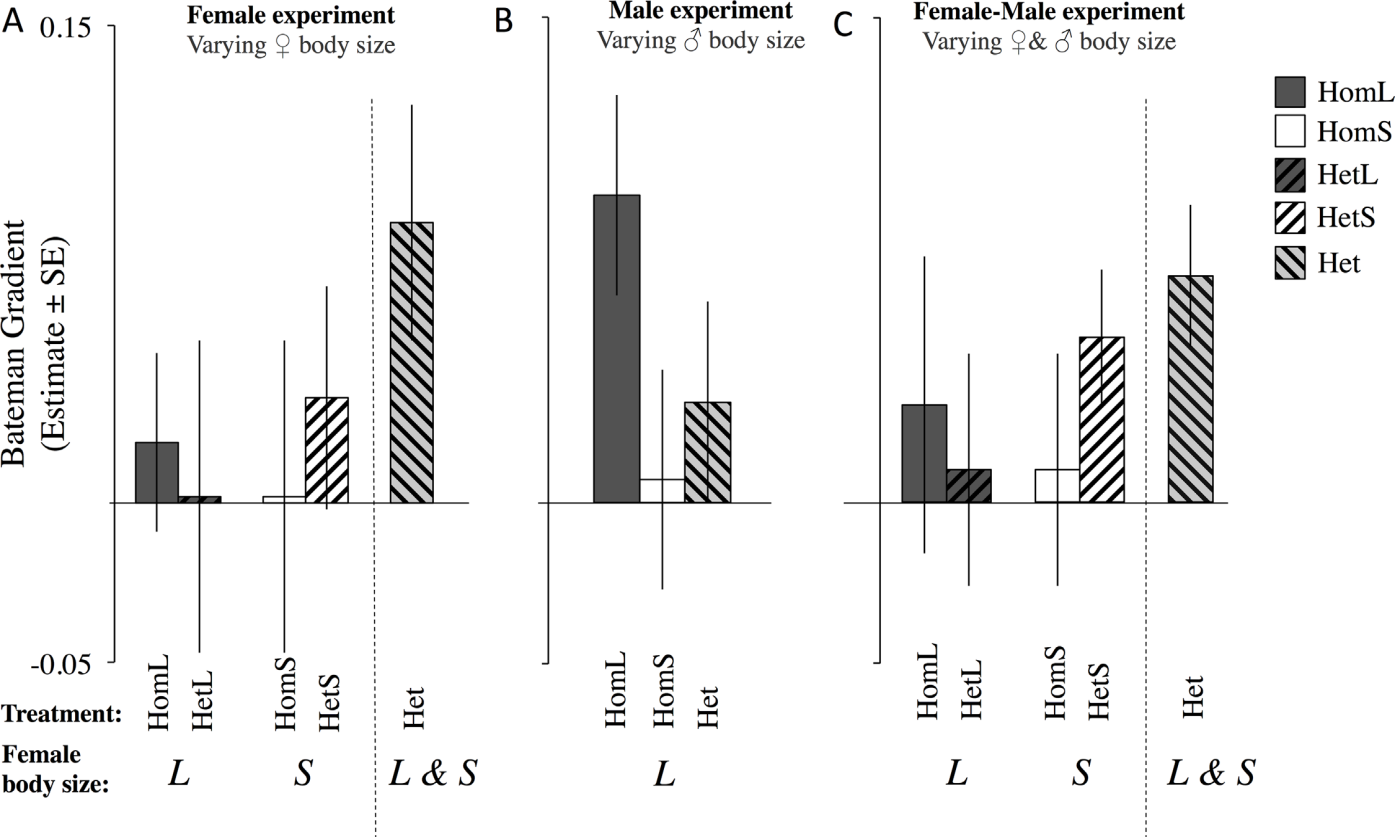
Female Bateman gradients. Estimate (± SE) extracted from the univariate models. Female Bateman gradients in (a) the Female Experiment; (b) the Male Experiment and (c) the Female -Male Experiment. Solid dark grey–Homogeneous Large, Solid white–Homogeneous Small, Dark grey striped from bottom left to upper right–Heterogeneous Large, White striped from bottom left to upper right–Heterogeneous Small, Light grey striped from bottom right to upper left–Heterogeneous (combined Large and Small).L–Large; S–Small.

Female Bateman gradients did not significantly differ from zero for most female groups, with the following exceptions. The two heterogeneous environments in which female body size varied were significantly positive (i.e. Het^F^ and Het^FM^, where both small and large females are considered as a single population). Also, HetS in the Female-Male Experiment (small females in the heterogeneous group, considered separately from large females), and HomL in the Male Experiment (large females with large males) were significantly positive (see Table 2 & S2 Table; Figs 3 & S2 and S1 Table).

**Table 2:** Female and focal male Bateman gradients. Estimate ± SE (p-value) are shown. Female estimates of the Bateman gradient are extracted from the univariate model. Male Bateman gradients, paternity share gradients and mate productivity gradients are extracted from the multivariate models. L–Large body size; S–Small body size. Bold–p-value ≤ 0.05. HomL–Homogeneous Large; HomS–Homogeneous Small; HetL–Heterogeneous Large, HetS–Heterogeneous Small, Het–Heterogeneous (combined large and small). The Female Experiment–varying female body size; The Male Experiment–varying male body size and the Female -Male Experiment–varying both female and male body size.

Test	Social manipulation	Factors	Sex analysed	Female experiment				Male experiment				Female-Male experiment			
				varying ♀ body size				varying ♂ body size				varying ♂ &♀ body size			
			* *	*Size*	*Estimate*	*SE*	*p-value*	*Size*	*Estimate*	*SE*	*p-value*	*Size*	*Estimate*	*SE*	*p-value*
*Social*	Hom Large	Mates	Female	L	0.019	0.028	0.496	L	0.112	0.034	**0.003**	L	0.031	0.051	0.538
*environment*		Vial ID			0.006	0.009	0.524		0.016	0.016	0.349		0.032	0.020	0.121
* *	Hom Small	Mates	Female	S	0.002	0.049	0.968	L	0.012	0.041	0.768	S	0.014	0.034	0.681
* *		Vial ID			-0.024	0.017	0.156		0.011	0.012	0.394		0.023	0.012	0.072
* *	Het	Mates	Female	L / S	0.098	0.040	**0.018**	L	0.024	0.031	0.455	L / S	0.072	0.026	**0.008**
* *		Vial ID			-0.001	0.006	0.785		-0.002	0.005	0.693		0.001	0.004	0.777
*Size within*	*Het Large*	Mates	Female	L	0.010	0.026	0.686	-	-	-	-	L	0.018	0.038	0.638
*Het groups*	* *	Vial ID			-0.001	0.004	0.694		-	-	-		0.008	0.006	0.186
* *	*Het Small*	Mates	Female	S	-0.004	0.039	0.913	-	-	-	-	S	0.047	0.026	0.077
* *	* *	Vial ID			-0.007	0.005	0.132		-	-	-		-0.005	0.003	0.131
*Social env*	Hom x Het Large	Mates * Social	Female	-	-0.001	0.047	0.770	-	-	-	-	-	-0.018	0.067	0.788
*within size*	Hom x Het Small	Mates * Social	Female	-	-0.015	0.057	0.786	-	-	-	-	-	0.035	0.046	0.451
* *															
*Social*	Hom Large	Mates	Male	L	0.104	0.060	0.146	L	-0.001	0.108	0.932	L	0.169	0.030	**0.004**
*environment*		Paternity			0.259	0.066	**0.011**		0.411	0.066	**0.008**		0.161	0.026	**0.003**
* *		Partner productivity			0.239	0.064	**0.014**		0.061	0.040	0.223		-0.018	0.037	0.636
* *		Vial productivity			0.000	0.000	0.730		0.000	0.000	0.965		0.000	0.000	0.300
		DF			NA	NA	-		0.075	0.219	0.062		0.168	0.091	0.139
* *	Hom Small	Mates	Male	L	0.822	0.245	**0.028**	S	0.134	0.047	**0.045**	S	0.339	0.037	**<0.001**
* *		Paternity			0.084	0.087	0.386		0.216	0.030	**0.002**		0.120	0.030	**0.016**
* *		Partner productivity			-0.149	0.159	0.402		0.093	0.027	**0.026**		0.211	0.034	**0.003**
* *		Vial productivity			-0.001	0.001	0.258		-0.001	0.000	**0.036**		0.001	0.000	**0.014**
		DF			NA	NA	-		0.143	0.083	0.160		-0.112	0.106	0.348
* *	Het	Mates	Male	L	0.038	0.029	0.205	L / S	0.082	0.043	0.081	L / S	0.231	0.033	**<0.001**
* *		Paternity			0.259	0.030	**<0.001**		0.295	0.044	**<0.001**		0.236	0.029	**<0.001**
* *		Partner productivity			0.004	0.026	0.875		0.138	0.041	**0.005**		0.046	0.026	0.108
* *		Vial productivity			0.000	0.000	**0.011**		0.000	0.000	0.148		0.000	0.000	0.169
		DF			-0.062	0.058	0.309		-0.177	0.085	0.056		-0.106	0.093	0.275
*Size within*	*Het Large*	Mates	Male	-	-	-	-	L	0.169	0.076	0.091	L	0.258	0.037	**0.002**
*Het groups*	* *	Paternity			-	-	-		0.197	0.188	0.354		0.252	0.020	**<0.001**
* *	* *	Partner productivity			-	-	-		0.149	0.217	0.529		0.097	0.072	0.247
* *	* *	Vial productivity			-	-	-		0.000	0.000	0.659		0.000	0.000	0.672
		DF							-0.127	0.268	0.66		-0.376	0.196	0.128
* *	*Het Small*	Mates	Male	-	-	-	-	S	0.025	0.067	0.726	S	0.257	0.047	**0.005**
* *	* *	Paternity			-	-	-		0.300	0.087	**0.025**		0.223	0.080	**0.050**
* *		Partner productivity			-	-	-		0.159	0.054	0.049		0.066	0.043	0.199
* *		Vial productivity			-	-	-		0.000	0.000	0.195		-0.001	0.000	0.066
		DF			-	-	-		-0.198	0.147	0.250		0.030	0.118	0.809
*Social env*	Hom x Het Large	Mates * Social	Male	-	-	-	-	L	0.137	0.196	0.501	L	0.020	0.045	0.659
*within size*		Paternity * Social			-	-	-		-0.287	0.188	0.161		0.069	0.037	0.090
* *		Partner productivity * Social			-	-	-		0.233	0.144	0.139		-0.061	0.054	0.283
* *		DF							0.119	0.117	0.333		0.106	0.080	0.252
* *	Hom x Het Small	Mates * Social	Male	-	-	-	-	S	-0.127	0.082	0.150	S	-0.179	0.076	**0.041**
* *		Paternity * Social			-	-	-		0.115	0.098	0.268		-0.052	0.100	0.613
		Partner productivity * Social			-	-	-		0.030	0.075	0.662		-0.257	0.067	**0.003**
		DF			-	-	-		-0.028	0.092	0.259		-0.052	0.118	0.667

We then tested whether the Bateman gradient of large and small females in heterogeneous social environments significantly differed from the Bateman gradient of large and small females in homogeneous social environments (Female and Female-Male Experiments), and found no significant differences (Fig 3, Table 2, S2 Table). The exclusion of a single female in the HomS^F^ group that mated but did not produce any offspring does not qualitatively change the results.

### Patterns of male reproduction

**Fig 4:**
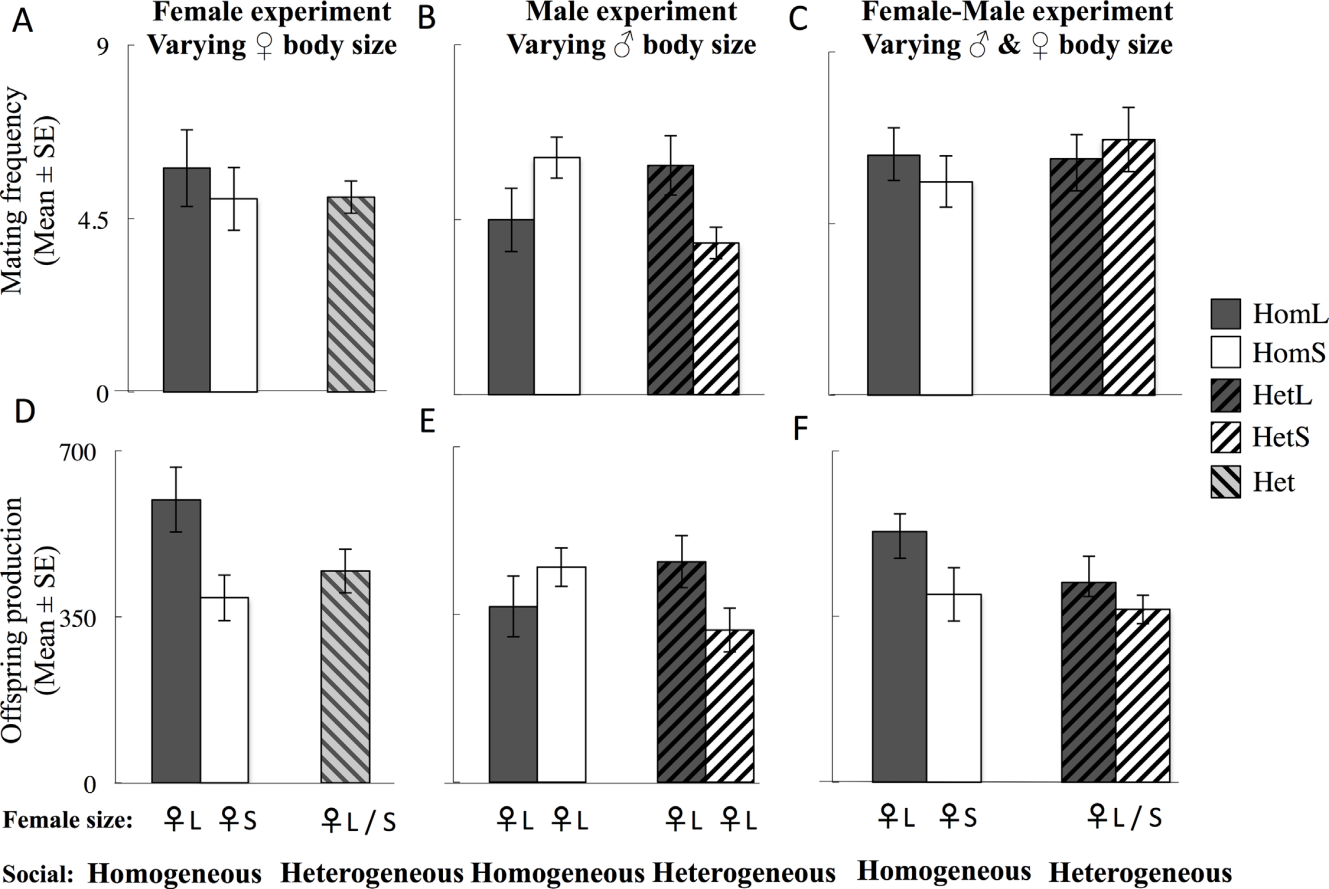
Effects of larval environment on focal male reproduction. **(a–c)** Mean mating frequency of the focal male: (a) Female Experiment; (b) Male Experiment and (c) Female-Male experiment. (d-f) The number of offspring sired by focal males: (a) Female Experiment; (b) Male Experiment and (c) Female-Male experiment. The size of females is represented below the barplot. ♀_L_—Large females; ♀_S_—Small females. Solid dark grey–Homogeneous Large, Solid white–Homogeneous Small, Dark grey striped from bottom left to upper right–Heterogeneous Large, White striped from bottom left to upper right–Heterogeneous Small, Light grey striped from bottom right to upper left–Heterogeneous (combined Large and Small).

The social environment affected the number of offspring sired by focal males in the Female Experiment with males producing more offspring in the HomL than in the Het followed by the HomS groups (post-hoc SNK test, **α** = 0.05), and in the Female-Male experiment with focal males in homogeneous groups producing more offspring than focal males in the heterogeneous group (Table 1, Fig 4D). Focal male mating frequency, number of mates or proportion of offspring sired across did not significantly differ with social environment in any experiment (Table 1, Fig 4A–4F, S4 Fig). In the Male Experiment, the proportion of offspring sired by large males (i.e. reared at low larval density) was significantly higher than for than small (high larval density) males (Table 1, S5 Fig). We found a significant interaction between social environment and focal male body size (i.e. social * body size) on focal male mating frequency, the proportion of offspring sired by focal males, and on the total number of offspring sired by focal males in the Male Experiment: small focal males in a heterogeneous social environment mated less frequently and sired fewer offspring than small males in a homogenous social environment (Table 1, Fig 4B and 4E, S5 Fig). We did not detect an effect of body size or the interaction body size and social environment in any of the focal male reproductive measures in the Female-Male Experiment (Table 1). The exclusion of one male in HomS^F^ and one male in HomL^M^ that failed to obtain any mates changes the analyses for number of offspring: the effect of social environment became non-significant in the Female Experiment (the p-value changed from 0.046 to 0.068) and, the interaction social * body size of the focal male on the number of offspring sired by males in the Male Experiment becomes marginally non-significant (p = 0.061) where it had previously been significant (p = 0.033).

### Sexual selection on males

Firstly, we applied Eq 3 to investigate the relative contribution of male mate number (*M)*, paternity share (*P*), mate productivity (*N*) and their covariances in explaining variance in offspring sired (*T*). Overall, variance in *P* explained the largest proportion of variance in *T*, followed by variance in *M* and the covariance between *M* and *P* (Table 3, see Methods). We then qualitatively investigated whether the social environment changed the relative contributions of *M*, *P*, *N* and their covariates. The decomposition of the variance in *T* for large and small focal male in a heterogeneous social environment had broadly the same characteristics of the decomposition of variance in *T* for focal males in a homogeneous social environment–i.e. variation in *T* was largely explained by variance in *P* followed by variation in *M* and the covariance between *M* and *P* (Table 3). A Commonality Analysis (CA), which uses an alternative method to decompose variance in *T*, qualitatively supports the overall contribution of variances and covariances related to *M*, *P* and *N* for male reproduction (S3 Table).

**Table 3:** Variance decomposition: observed contributions of the variances and covariances in *M*, *N* and *P* in explaining the variance in the number of offspring sired by males (*T*). The decomposition is based on the approach proposed by Webster, PruettJones [15]. *Var(x)* represents the variance component of the factor *x* (i.e. *M*, *N or P*), and *cov(x*, *y)* represents the covariance components between the factors *x* and *y* (e.g. covariance between *M* and *P*–cov(*M*, *P*)). *D* represents the variance in the error term ε. HomL–Homogeneous Large; HomS–Homogeneous Small; HetL–Heterogeneous Large, HetS–Heterogeneous Small, Het–Heterogeneous. The Female Experiment–varying female body size; The Male Experiment–varying male body size and The Female-Male Experiment–varying both female and male body size.

Var-Cov Components	*Observed contribution to var(T)*																									
	Female experiment						Male experiment										Female-Male experiment									
	*HomL*	*%*	*HomS*	*%*	*Het*	*%*	*HomL*	*%*	*HomS*	*%*	*HetL*	*%*	*HetS*	*%*	*Het*	*%*	*HomL*	*%*	*HomS*	*%*	*HetL*	*%*	*HetS*	*%*	*Het*	*%*
***var(T)***	47.14		46.03		21.34		40.32		16.20		29.50		20.04		29.00		14.77		32.26		31.22		9.17		19.39	
*var(M)*	13.52	28.67	17.20	37.36	4.37	20.48	17.78	44.09	4.59	28.36	11.04	37.42	4.31	21.50	6.96	23.99	10.51	71.14	5.16	15.98	13.96	44.72	11.00	119.96	11.84	61.05
*var(P)*	15.17	32.18	10.22	22.21	12.36	57.92	17.48	43.35	4.88	30.10	12.43	42.15	10.15	50.64	16.69	57.57	8.25	55.84	6.92	21.45	18.76	60.09	6.62	72.14	11.85	61.12
*var(N)*	5.46	11.59	4.61	10.01	3.49	16.34	0.42	1.03	1.76	10.85	2.38	8.08	1.89	9.42	2.25	7.76	0.99	6.67	2.16	6.70	0.44	1.42	1.69	18.47	1.01	5.22
*cov(M*, *P)*	4.84	10.26	-0.59	-1.28	9.21	43.16	18.75	46.49	0.73	4.49	11.03	37.39	5.81	29.00	8.69	29.95	-3.19	-21.57	-2.65	-8.20	17.40	55.74	6.24	68.02	11.24	57.96
*cov(M*, *N)*	5.08	10.78	9.80	21.29	0.66	3.08	1.94	4.82	0.92	5.70	2.38	8.08	1.85	9.24	2.02	6.97	3.06	20.73	-2.68	-8.31	1.44	4.62	-2.72	-29.67	-0.60	-3.07
*cov(N*, *P)*	-5.42	-11.50	1.81	3.94	2.39	11.22	2.25	5.59	2.52	15.56	8.97	30.42	0.82	4.11	3.74	12.90	-0.34	-2.31	1.19	3.67	-0.17	-0.55	-4.26	-46.43	-2.09	-10.78
*D*	8.50	18.02	2.98	6.47	-11.14	-52.20	-18.29	-45.37	0.80	4.93	-18.74	-63.53	-4.79	-23.91	-11.35	-39.14	-4.51	-30.49	22.17	68.71	-20.62	-66.04	-9.40	-102.50	-13.86	-71.49

**Fig 5:**
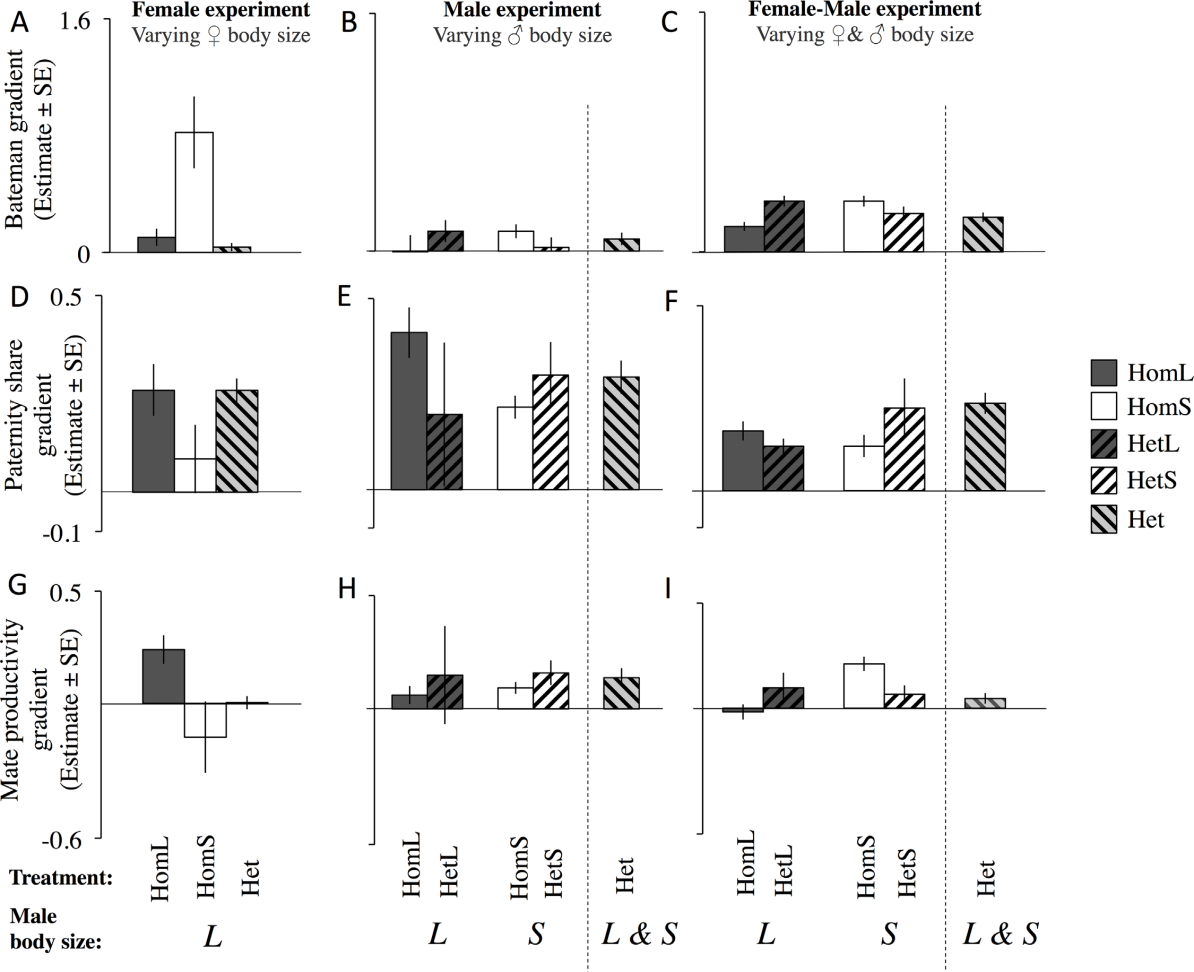
Focal male Bateman gradients, paternity share gradients and mate productivity gradients. Estimate (±SE) extracted from the multivariate models. (a-c) the focal male Bateman gradients in (a) the Female Experiment; (b) the Male Experiment and (c) the Female-Male Experiment. (d-f) paternity share gradients in (d) the Female Experiment; (e) the Male Experiment and (f) the Female-Male Experiment. (g-i) mate productivity gradients in (g) the Female Experiment; (h) the Male Experiment and (i) the Female-Male Experiment. Solid dark grey–Homogeneous Large, Solid white–Homogeneous Small, Dark grey striped from bottom left to upper right–Heterogeneous Large, White striped from bottom left to upper right–Heterogeneous Small, Light grey striped from bottom right to upper left–Heterogeneous (combined Large and Small). L–Large; S–Small.

We then used a predictive multivariate linear model (Eq 4) to test whether the slope of the gradient of a focal male’s offspring number regressed over mate number (*M)*, paternity share (*P)*, and mate productivity gradients (*N)* controlling for the other gradients were influenced by our social manipulations. First, we tested whether the focal male multivariate *M*, *P* and *N* gradients were significantly different from zero in the homogenous and heterogeneous groups. Significantly positive multivariate *M* gradients (Bateman gradients) were observed for large males in the HomS^F^ group of the Female Experiment (where large males were exposed to small females), for small males in the HomS^M^ group of the Male Experiment (where small males were exposed to large females), and in all groups of the Female-Male Experiment (i.e. HomL^FM^, HomS^FM^ and Het^FM^; Table 2; Fig 5A–5I). However, multivariate *M* gradients were positive but not significantly different from zero in the HomL^F^ group of the Female Experiment (where large males were exposed to large females), in the Het^F^ group of the Female Experiment (where large males were exposed to females of mixed body sizes), and in the Het^M^ group of the Male Experiment (where mixed size males were exposed to large females) (Fig 5A, Table 2, S6 Fig). Multivariate *M* gradient did not show a positive trend in the HomL^M^ group of the Male Experiment (where large males were exposed to large females; see Fig 5A, Table 2, S6 Fig). Multivariate *P* gradients were highly significant in all social environment manipulations except HomS^F^ (Table 2). Significantly positive multivariate *N* gradients were limited to HomL^F^, HomS^M^, Het^M^, and HomS^FM^ (Table 2).

We next investigated whether *M*, *P* and *N* gradients were altered when focal males of different body sizes experienced a heterogeneous versus a homogenous social environment. We did not find a significantly greater than zero *M* gradients for either large or small focal males in a heterogeneous environment of the Male Experiment (i.e. HetL^M^ and HetS^M^). However, the *M* gradient was significantly greater than zero for both large and small focal males of the Female-Male Experiment (i.e. HetL^FM^ and HetS^FM^; Table 2, S7 Fig). *P* gradient was significantly positive for small focal males but not large focal males in the heterogeneous environment of the Male Experiment, and was significantly positive for both large and small focal males in a heterogeneous environment of the Female-Male Experiment (Table 2). Multivariate *N* gradients were not significantly different from zero for small or large males in any of the heterogeneous groups (Table 2).

We then compared *M*, *P* and *N* gradients of large and small males in a heterogeneous environment (considering each size class separately) with the gradients of large and small males in a homogeneous environment, respectively. We found for small focal males that both *M* and *N* gradients were strongly reduced in a heterogeneous social environment of the Female-Male Experiment (HetS^FM^) relative to small males in a homogeneous social environment (Fig 5C and 5I & S7 Fig, Table 2 & S2 Table). These effects were not observed for either small focal males of the Male Experiment (HetS^M^) or large focal males of both Male and Female-Male Experiments, where *M*, *P* or *N* did not significantly differ between heterogeneous and homogeneous social environments (Table 2, S2 Table).

## Discussion

- We confirmed our predictions that larval density generate large body sized individuals that are more successful in obtaining mates and matings as well as producing offspring.
- We confirmed that the positive relationship between the number of mates and offspring of females arise from differences in female body size (and total productivity) in a group. This result highlights the potential for misleading interpretations of a positive female Bateman gradient.
- We partially confirmed our expectations of an overall positive Bateman gradient for both large and small males, although this was not true for all treatments.
- We found that, in a mixed environment where both sexes varied in body size, neither female body size nor social environment affected the number of offspring *per* mating produced by females. This finding may open a new perspective on the evolution of male mate choice by showing that males do not necessarily gain additional offspring from mating with large (more fecund) females.
- We also found that small, but not large focal males have a significantly reduced Bateman gradient and partner fecundity gradient in a mixed social environment when both sexes varied in body size, suggesting that small individuals suffer costs from competing with large individuals.

Below, we discuss the main findings of our study in detail.

### Sexual selection in females

Our data suggest that the larval environment experienced by individuals within groups can influence adult Bateman gradients in female *D*. *melanogaster*. For instance, we found that the Bateman gradients were significantly positive in heterogeneous social environments whereas gradients were less steep, and not significantly different from 0 in most (though not all) homogenous social environments (Table 2, Fig 3). As predicted, the steep Bateman gradients in heterogeneous social environments likely arise as a consequence of associations between larval density effects on the number of offspring produced by adult females (likely linked to body size), and their mating frequency. As expected, females grown at high larval density eclose smaller, produce fewer offspring, and have lower mating frequencies relative to females grown at low density (i.e. large adult females). Thus, in populations containing both large and small females, there is inevitably a correlation between mate number and number of offspring produced, which is ultimately driven by differences in the larval environments. This idea is supported by the fact that, when we compared the heterogeneous social environment Bateman gradients for small and large females considered separately, we did not see significant differences from small and large females in homogeneous social environments. This suggests that it is the heterogeneity of females *per se*, not changes in selection on small and large females that generates the positive Bateman gradients. In other words, the positive female Bateman gradients in heterogeneous groups are an emergent property of the heterogeneity in larval conditions experienced by female individuals within the group. Our results therefore highlight the fact that Bateman gradients should be interpreted cautiously as a measure of sexual selection on mate number in females, because the correlation between number of offspring produced and number of mates does not necessarily reflect causality [12, 16, 18, 24, 29, 30, 68, 69]. We also saw unexplained variation in Bateman gradients between experiments. For example in the HomL group of the Male Experiment the Bateman gradient was significantly different from 0, but in the Female or Female-Male experiments HomL was much less steep, and not significantly different from 0, despite the fact that all HomL groups were treated identically. Thus, we should exhibit some caution when interpreting the data.

### Sexual selection in males

Our results suggest a complex architecture of male reproductive success, and highlight the importance of decomposing the offspring number sired by males into its *M*, *P* and *N* components [6, 11–13, 28]. For example, the covariance between *M* and *P* was mostly positive across experiments (except in the HomL^FM^ and HomS^FM^), which suggests that males that mate with more females obtain higher paternity share with their mates (Table 3). Conversely, a negative covariance between *M* and *P* (as in HomS^FM^ and HomL^FM^) reflects a potential trade-off between pre- and post-copulatory success, in which males that attract more mates are poor post-copulatory competitors (Table 3). Whilst males are expected to gain from additional mates, the primary mechanisms by which males gain fitness might in some cases be via mate choice for high productivity females (but see below), or via success in post-copulatory competition, and these traits may or may not be correlated with pre-copulatory success (Table 2) [11, 12, 70]. Our finding that *P* explains a large proportion of variance is broadly consistent with the findings in other promiscuous species such as the red jungle fowl *Gallus gallus* [11, 12], guppies *Poecilia reticulata* [71] and the hermaphrodite snail *Physa acuta* [28]. However, it is important to interpret our results for *P* with caution. For instance, in a study on semelparous-adapted *D*. *melanogaster* population–which was cultured each generation with a single short reproductive bout–most post-copulatory success was explained by last male sperm precedence, leaving only a further ~2% of variance in reproductive success explained by additional post-copulatory processes [72, 73]. This previous study show that last male precedence can significantly contribute to the overall strength of post-copulatory sexual selection, and could be a confounding effect when analysing post-copulatory processes (*P)*. Other mechanisms that might also contributes to variance in *P* include cryptic female choice [74] and sperm viability [26, 75, 76], which skew male fertilization success similar to male precedence. Interestingly, however, Pischedda and Rice [73] showed that the total variance in male reproductive success explained by either general post-copulatory processes or by post-copulatory processes after controlling for male precedence was the same, suggesting that male precedence is a component of, instead of additional to, *P* (see Fig 2 in [73]). Thus, it is unlikely that considering male precedence (or other processes) would explain additional variance than that already captured by *P* and, consequently, it would not alter the relative contributions of *M* and *N*. Therefore, although male precedence, female cryptic choice and sperm viability are important, their relative contributions to the variance in *P* lie outside the scope of this paper and should be the focus of future analysis. It will also be particularly informative for future studies to determine how much last-male sperm precedence explains post-copulatory success in other iteroparous populations.

We found that small males in groups consisting of both males and females from different larval densities (i.e. the heterogeneous group in the Female-Male Experiment.) had significantly reduced Bateman and mate productivity gradients relative to small males in the homogeneous social environment (Table 2, Fig 5). This suggests that the operation of sexual selection–the benefits of obtaining multiple mates, and obtaining mates of high productivity–are modified for small males when in the presence of large males and a mix of female phenotypes. This is likely explained by a combination of factors. When in competition with large males, small males have a lower share of paternity because they are outcompeted over access to females and possibly because females favour inseminations by large males during or after mating. Such loss of paternity would erode the benefits of mating with multiple females [77], thus weakening the Bateman gradient of small males. These factors may also mean that small males may have a relatively higher share of paternity with smaller, less fecund females leading to weaker gradients of sexual selection on mate productivity and the large negative covariance between *N* and *P* found in small males in the HetS^FM^ treatment (Table 3).

Our results–and previous studies–show that *D*. *melanogaster* males who experience favourable developmental environments, that lead to large adult size, display higher success in pre and post-copulatory competition (Fig 4A–4C, Table 2) [30, 46]. Large adult males can obtain higher proportion of offspring for several reasons: they may be able to either monopolize most of the mates, elicit mating behaviour more frequently or effectively [46], win sperm competition, or be preferred in cryptic female choice [29]. Moreover, the larval developmental environment influences ejaculate investment, whereby males with small body size invest relatively more ejaculate to each mating relative to large males [36]. Thus, small males experiencing a heterogeneous environment might have adjusted their behaviour to accommodate the competition with large males, leading to an alternative path to reproductive success.

Variation in female productivity is expected to be a major driving force in the evolution of male mate choice, and current theory predicts that male mate choice for large adult females should evolve because large females produce more eggs (reviewed by Bonduriansky [26]). In *D*. *melanogaster*, some studies indicate that males preferentially court large, highly fecund females [43, 48] to gain fitness as a consequence of mate choice [78]. However, other studies suggest that male *D*. *melanogaster* can prefer small females, raising questions over the consistency of male preferences [78, 79]. We found that the number of offspring that a female produced *per* mating was not higher for large (low larval density) females when both sexes varied in size (The Female -Male Experiment, Fig 2G–2I). Large females remate more frequently [29, 36] meaning that sperm competition may be more intense for mates of large females, potentially offsetting the benefits of mating with high fecundity large females. The positive correlation between developmental environment-induced changes in body size and productivity [26] may mean that large females are more receptive to mating simply because they require greater quantities of ejaculate in order to fertilize the large number of eggs that are produced [80]. Similarly, large females may have lower sensitivity to male receptivity-inhibiting seminal proteins resulting in a more rapid return to receptivity. In addition, the low larval density might have served as cue of low mating opportunities, hence priming females to have higher receptivity (or lower resistance) to matings [36]. Males might prefer and target large females simply because large females are more willing to remate, or represent larger and slower targets for courtship. The idea that males do not always gain an advantage by mating with large females has received empirical support in the golden-orb web spider *Neuphila plumipes*, in which males changed their mate preference as the levels of intrasexual competition increased, but not necessarily targeted larger females [81]. Thus, male mate preference can be thought of as analogous to the Ideal-Free Distribution Model (IFD) where females are seen as patches and males tend to distribute themselves according to “female’s quality” [81]. Therefore, male choice for large females might arise because body size is correlated with the rate of egg production, not just total egg production, in which fast reproduction is likely beneficial in stable and expanding populations [35, 82].

In conclusion, our results suggest that the developmental environment can influence the operation of sexual selection during adulthood. Our study also adds to the growing body of evidence that shows the importance of considering more than simply the number of mates and offspring in measures of the strength of sexual selection on males [11–13]. Key questions for future studies include 1) how commonly does variation in female developmental environment or adult condition generate positive associations between mate and offspring number? 2) Do males often gain similar numbers of progeny per mating from low and high condition females? 3) How commonly do environmental conditions influence the benefits of additional mates for males? 4) To what extent do mechanisms other than mate number (e.g. paternity share) explain variation in the number of offspring sired by males?

## Supporting Information

S1 Fig Mean number of mates of females. (a) The Female Experiment; (b) The Male Experiment; (c) The Female-Male Experiment. Error bars = ±SE. Solid dark grey–Homogeneous Large, Solid white–Homogeneous Small, Dark grey striped from bottom left to upper right–Heterogeneous Large, White striped from bottom left to upper right–Heterogeneous Small, Light grey striped from bottom right to upper left–Heterogeneous (combined Large and Small).
(TIFF)

S2 Fig (a-c) **Univariate Bateman gradients of females**. (a) The Female Experiment; (b) The Male Experiment and (c) the Female-Male Experiment. Homogeneous Large (HomL), Homogeneous small (HomS) and Hetergoeneous (Het) groups.
(TIFF)

S3 Fig Female Bateman gradients of the heterogeneous group in which female varied in body size (Famale and Female-Male Experiments). Purple–Large body size; Blue–Small body size; Dashed line–Univariate Bateman gradient of the whole experimental treatment. (a) The Female Experiment, varying female body size. (b) The Female-Male Experiment, varying male and female body size.
(TIFF)

S4 Fig Mean number of mates of focal males. (a) The Female Experiment; (b) The Male Experiment; (c) The Female-Male Experiment. Means ±SE are shown. Solid dark grey–Homogeneous Large, Solid white–Homogeneous Small, Dark grey striped from bottom left to upper right–Heterogeneous Large, White striped from bottom left to upper right–Heterogeneous Small, Light grey striped from bottom right to upper left–Heterogeneous (combined Large and Small).
(TIFF)

S5 Fig Mean proportion of focal male’s offspring production. (a) The Female Experiment; (b) The Male Experiment; (c) The Female-Male Experiment. Means ±SE are shown. Solid dark grey–Homogeneous Large, Solid white–Homogeneous Small, Dark grey striped from bottom left to upper right–Heterogeneous Large, White striped from bottom left to upper right–Heterogeneous Small, Light grey striped from bottom right to upper left–Heterogeneous (combined Large and Small).
(TIFF)

S6 Fig Focal male univariate Bateman gradients. (a) The Female Experiment; (b) The Male Experiment and (c) The Female-Male Experiment. Homogeneous Large (HomL), Homogeneous small (HomS) and Hetergoeneous (Het) groups.
(TIFF)

S7 Fig Focal male’s Bateman gradient of the Heterogeneous group in which male (the Male Experiment), or both sexes varied in body size (the Female-Male Experiment). Purple–Large body size; Blue–Small body size; Dashed line–Univariate Bateman gradient. (a) The Male Experiment (b) The Female-Male Experiment; Small (blue)–Large (purple).
(TIFF)

S1 Table Univariate analysis of male Bateman gradients. Bold–p–value ≤ 0.1.
(XLSX)

S2 Table Comparison of the selection gradients of individuals with different body sizes in homogenous or heterogeneous social environments. Estimates ± SE (*t- value* and p-values) are shown. Bold–*t*-statistics p–value ≤ 0.1. *M–*number of mates, *P–*paternity share, *N–*mate productivity. HomL–Homogeneous Large; HomS–Homogeneous Small; HetL–Heterogeneous Large, HetS–Heterogeneous Small, Het–Heterogeneous. The Female Experiment–varying female body size; The Male Experiment–varying male body size and the Female-Male Experiment–varying both female and male body size.
(XLSX)

S3 Table Commonality analysis (CA) to partition the explained variance in the number of offspring sired by males. The results are given in percentage of explained variance. *SEC–*social environment composition (i.e. homogeneous vs. heterogeneous). *M*–number of mates, *P–*paternity share, *N-* mate productivity, *VP–*vial productivity. HomL–Homogeneous large, HomS–Homogeneous small, HetL–Heterogeneous large, HetS–Heterogeneous small, Het–Heterogeneous.
(XLSX)

S1 Text Supplementary_Results. Analyses of the effects of colour and changes of sex ratio due to female deaths.
(DOCX)
