# Supplementary figures and images for: Developmental Environment Effects on Sexual Selection in Male and Female Drosophila melanogaster
Source: PLoS One. 2016 May 11;11(5):e0154468. doi: 10.1371/journal.pone.0154468 (PMC4864243; doi:10.1371/journal.pone.0154468)

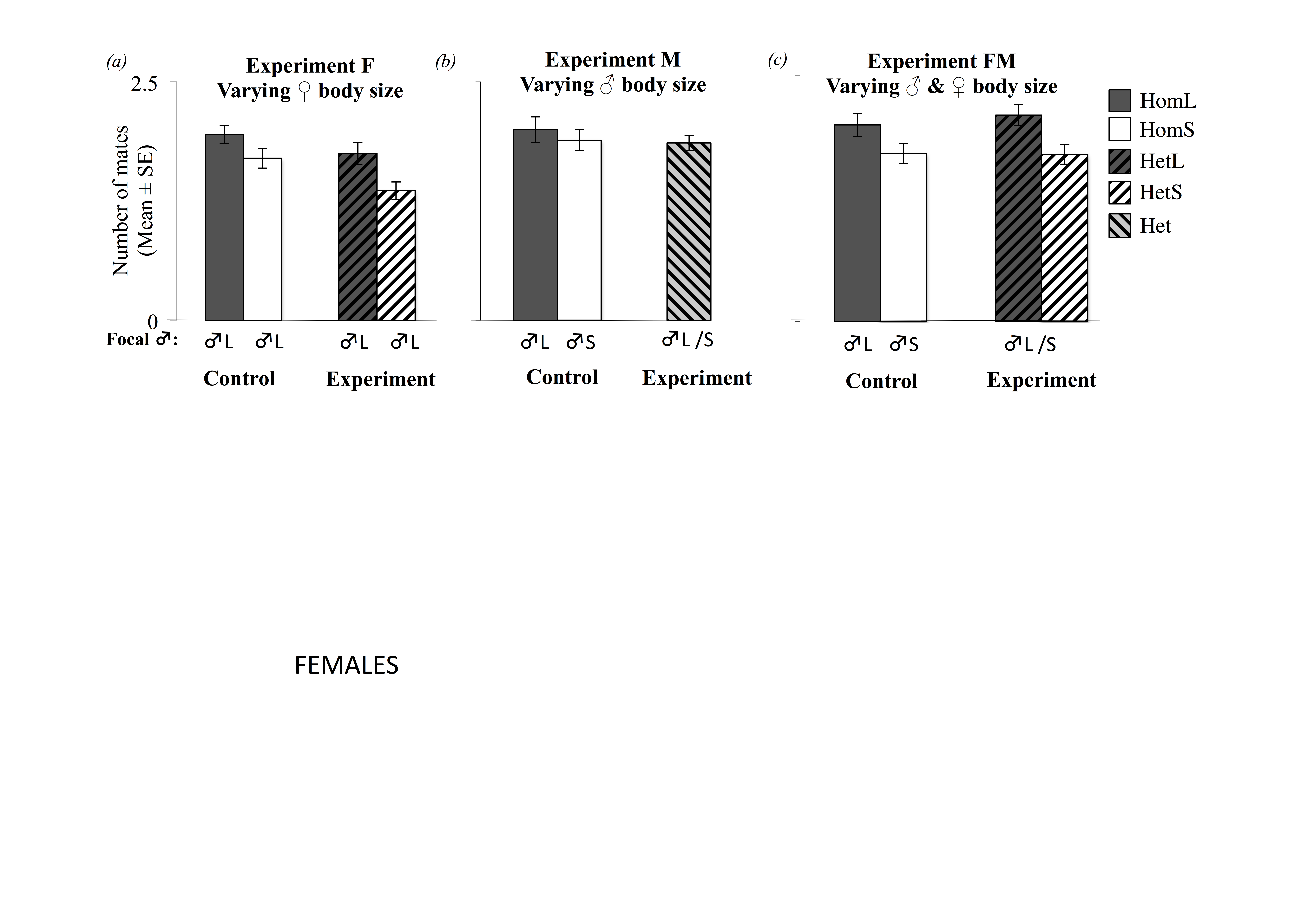

Supplement: S1 Fig — (a) The Female Experiment; (b) The Male Experiment; (c) The Female-Male Experiment. Error bars = ±SE. Solid dark grey–Homogeneous Large, Solid white–Homogeneous Small, Dark grey striped from bottom left to upper right–Heterogeneous Large, White striped from bottom left to upper right–Heterogeneous Small, Light grey striped from bottom right to upper left–Heterogeneous (combined Large and Small). (TIFF) [file pone.0154468.s001.tiff]

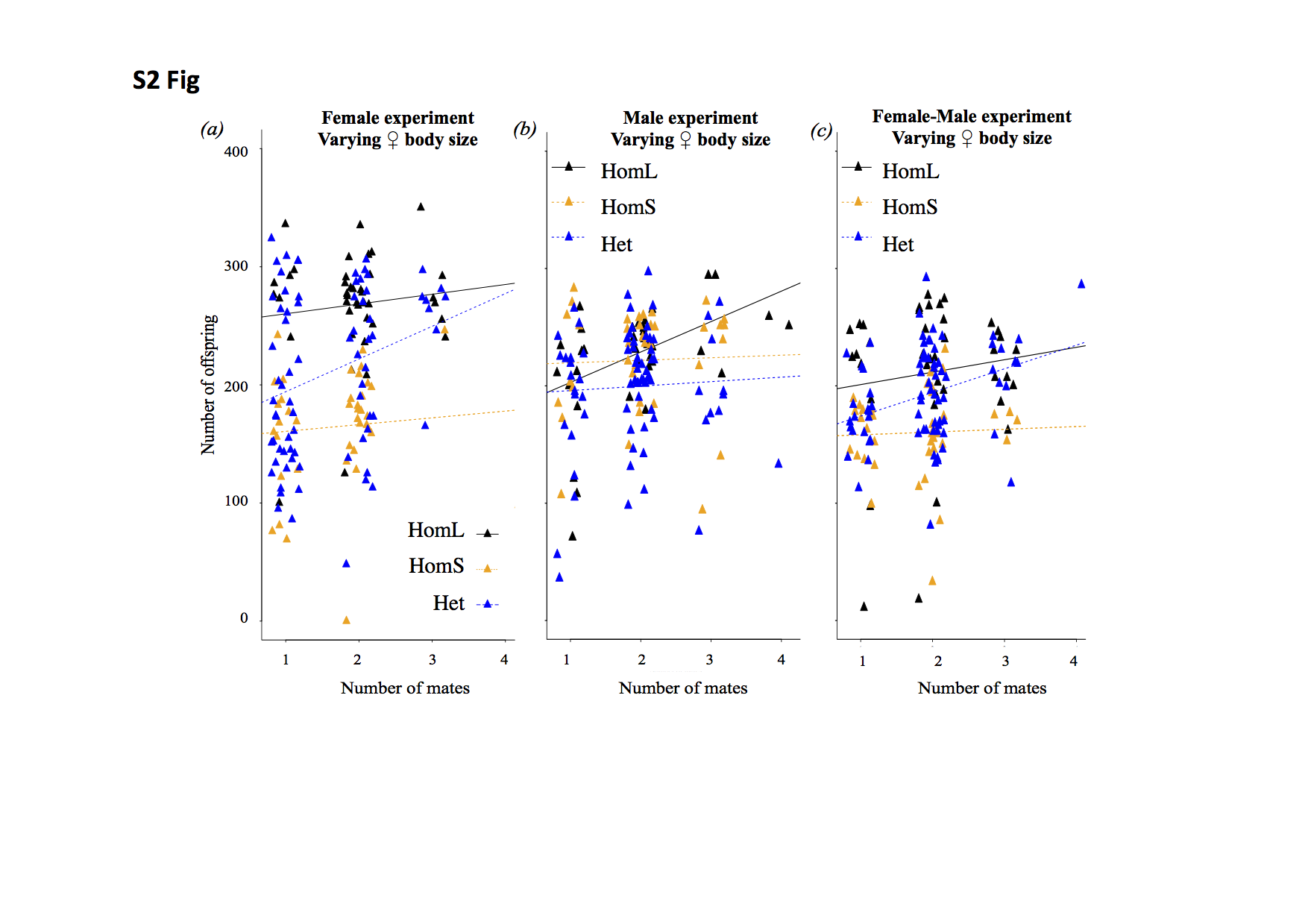

Supplement: S2 Fig — (a-c) Univariate Bateman gradients of females. (a) The Female Experiment; (b) The Male Experiment and (c) the Female-Male Experiment. Homogeneous Large (HomL), Homogeneous small (HomS) and Hetergoeneous (Het) groups. (TIFF) [file pone.0154468.s002.tiff]

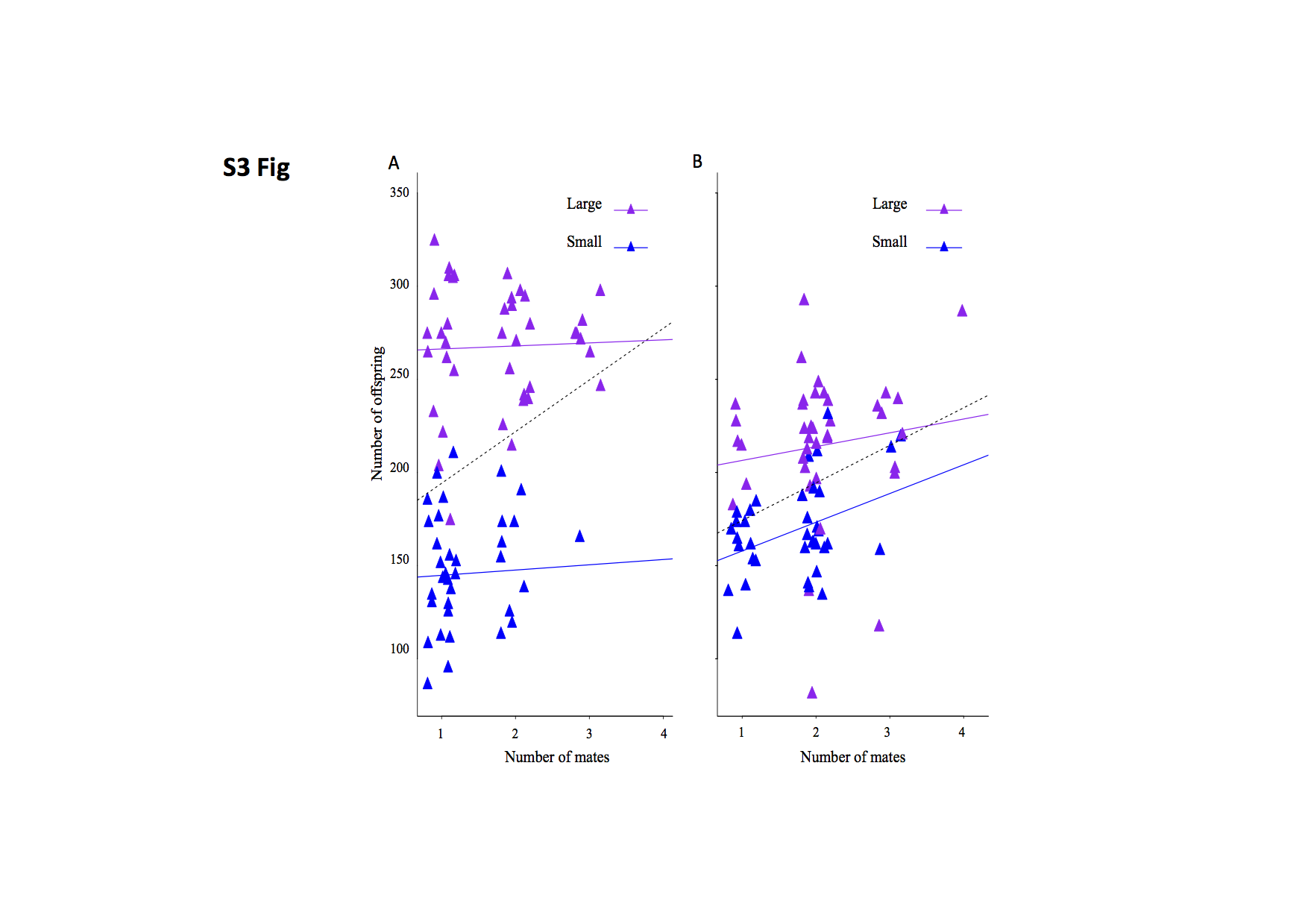

Supplement: S3 Fig — Purple–Large body size; Blue–Small body size; Dashed line–Univariate Bateman gradient of the whole experimental treatment. (a) The Female Experiment, varying female body size. (b) The Female-Male Experiment, varying male and female body size. (TIFF) [file pone.0154468.s003.tiff]

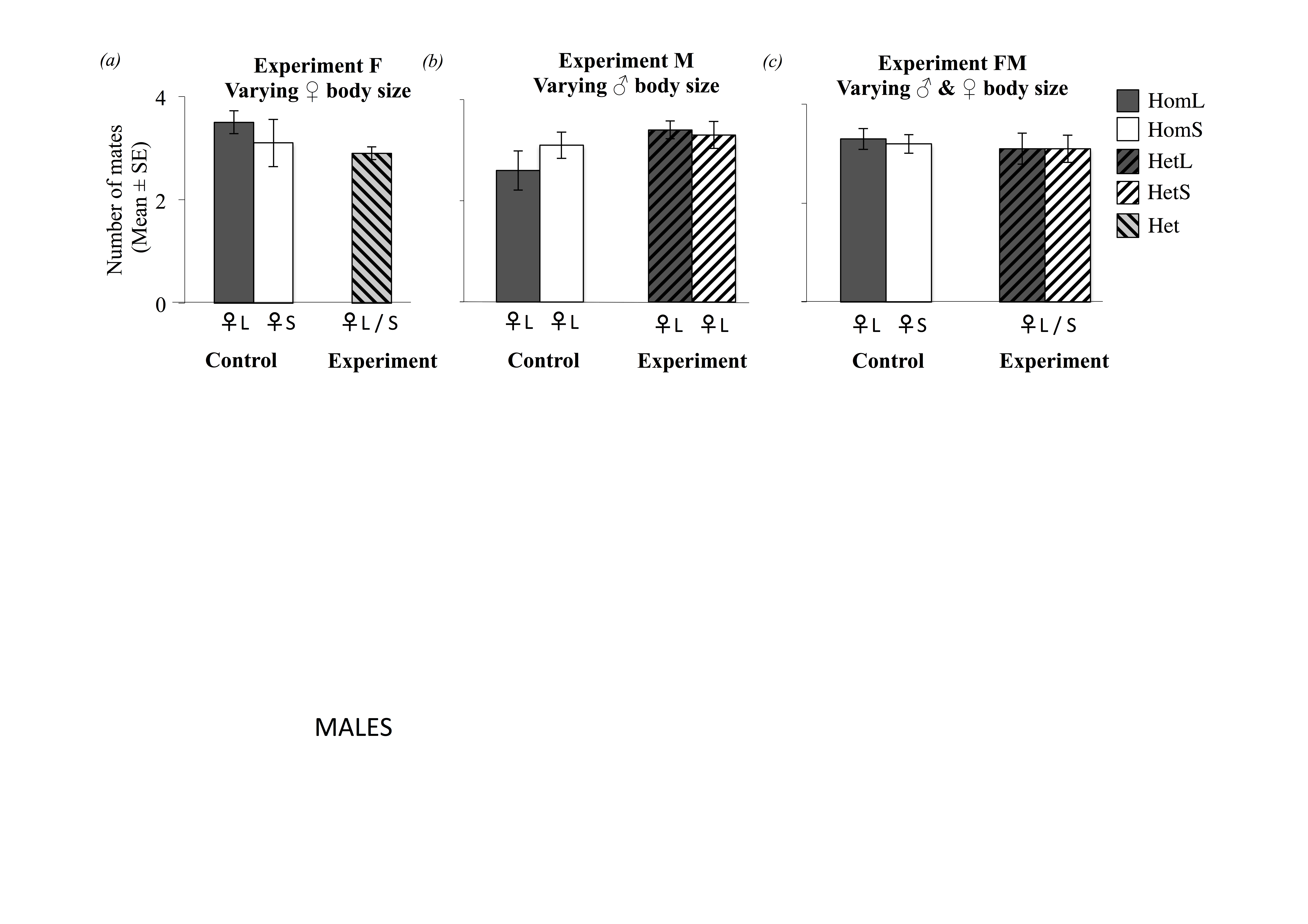

Supplement: S4 Fig — (a) The Female Experiment; (b) The Male Experiment; (c) The Female-Male Experiment. Means ±SE are shown. Solid dark grey–Homogeneous Large, Solid white–Homogeneous Small, Dark grey striped from bottom left to upper right–Heterogeneous Large, White striped from bottom left to upper right–Heterogeneous Small, Light grey striped from bottom right to upper left–Heterogeneous (combined Large and Small). (TIFF) [file pone.0154468.s004.tiff]

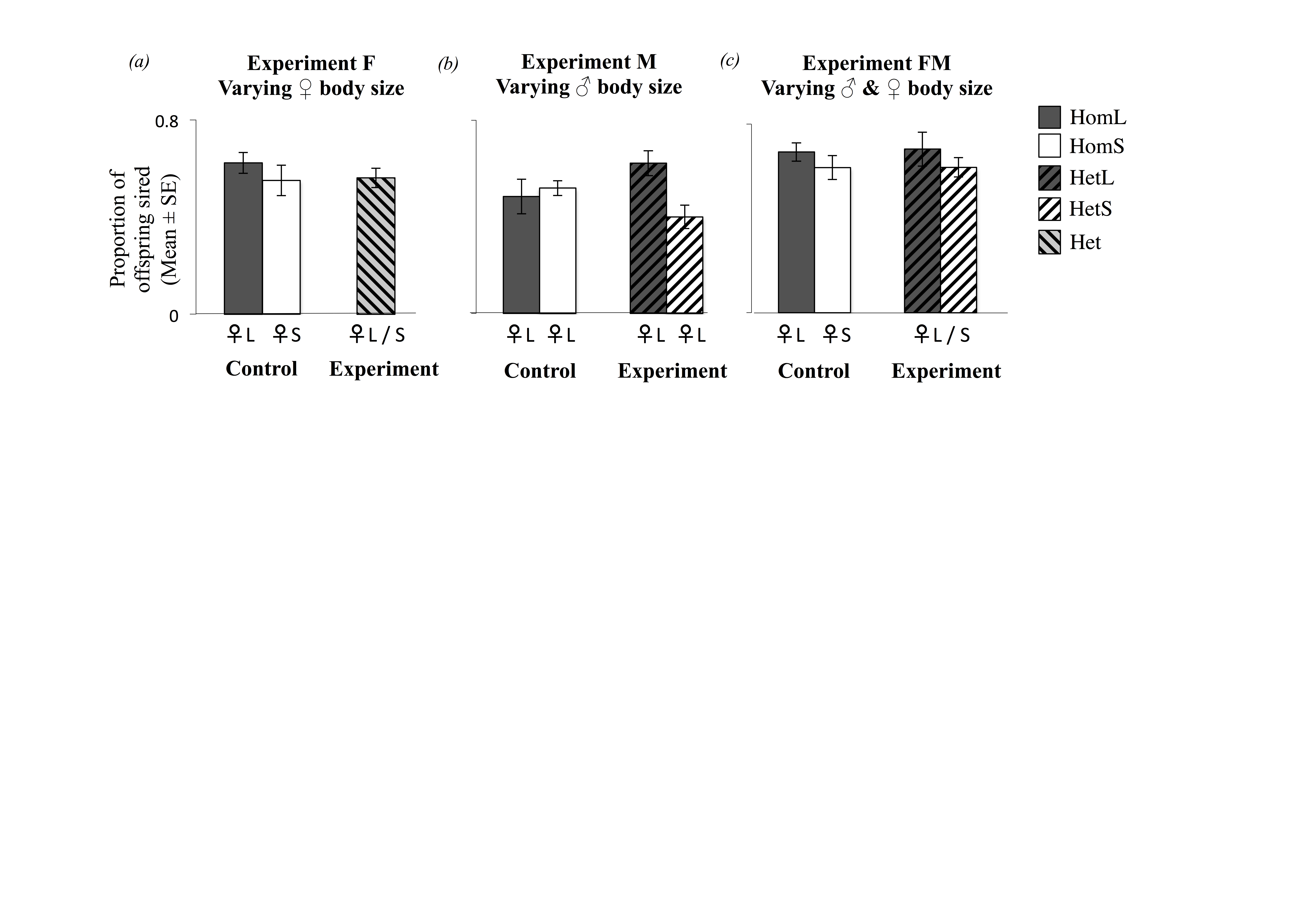

Supplement: S5 Fig — (a) The Female Experiment; (b) The Male Experiment; (c) The Female-Male Experiment. Means ±SE are shown. Solid dark grey–Homogeneous Large, Solid white–Homogeneous Small, Dark grey striped from bottom left to upper right–Heterogeneous Large, White striped from bottom left to upper right–Heterogeneous Small, Light grey striped from bottom right to upper left–Heterogeneous (combined Large and Small). (TIFF) [file pone.0154468.s005.tiff]

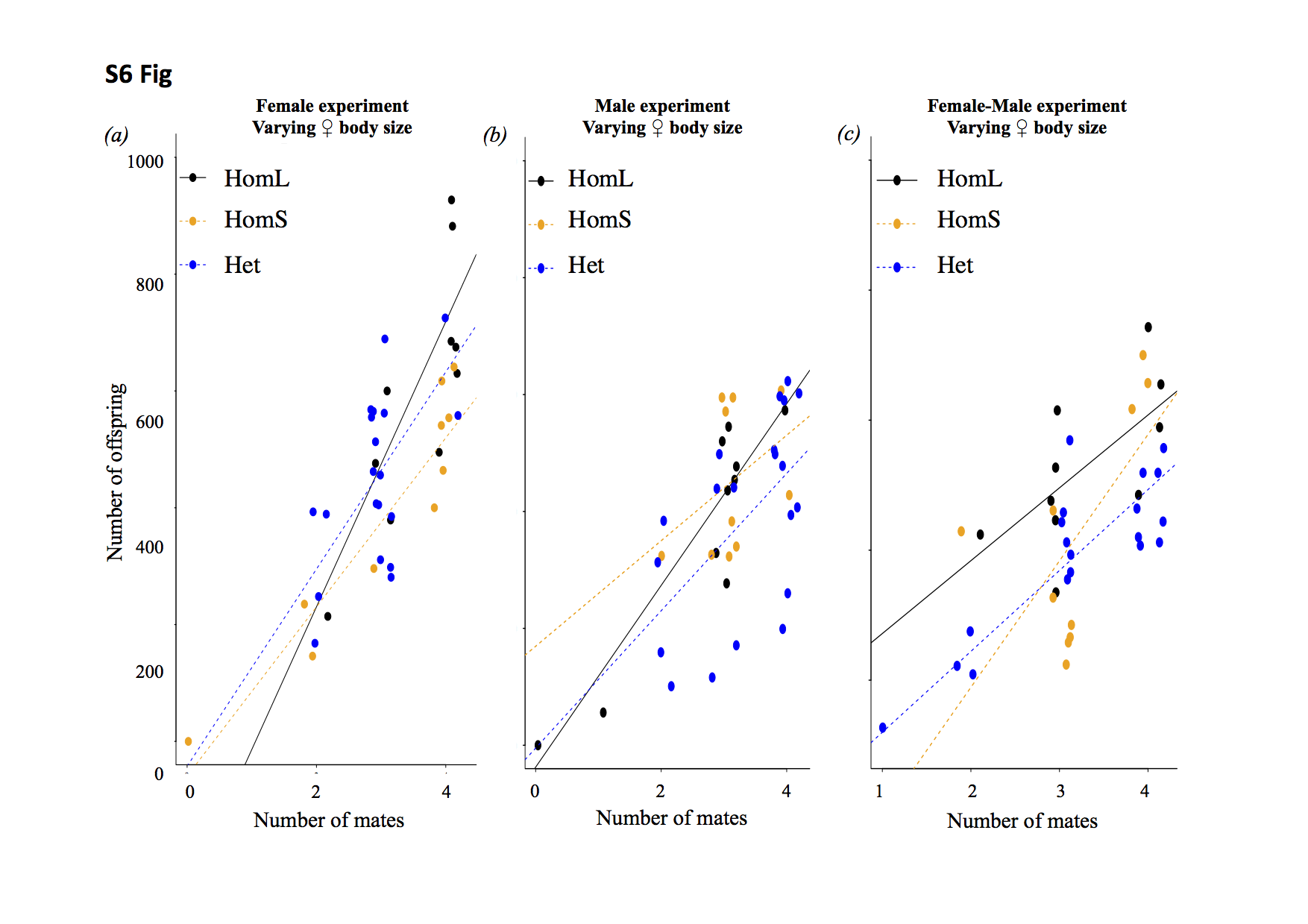

Supplement: S6 Fig — (a) The Female Experiment; (b) The Male Experiment and (c) The Female-Male Experiment. Homogeneous Large (HomL), Homogeneous small (HomS) and Hetergoeneous (Het) groups. (TIFF) [file pone.0154468.s006.tiff]

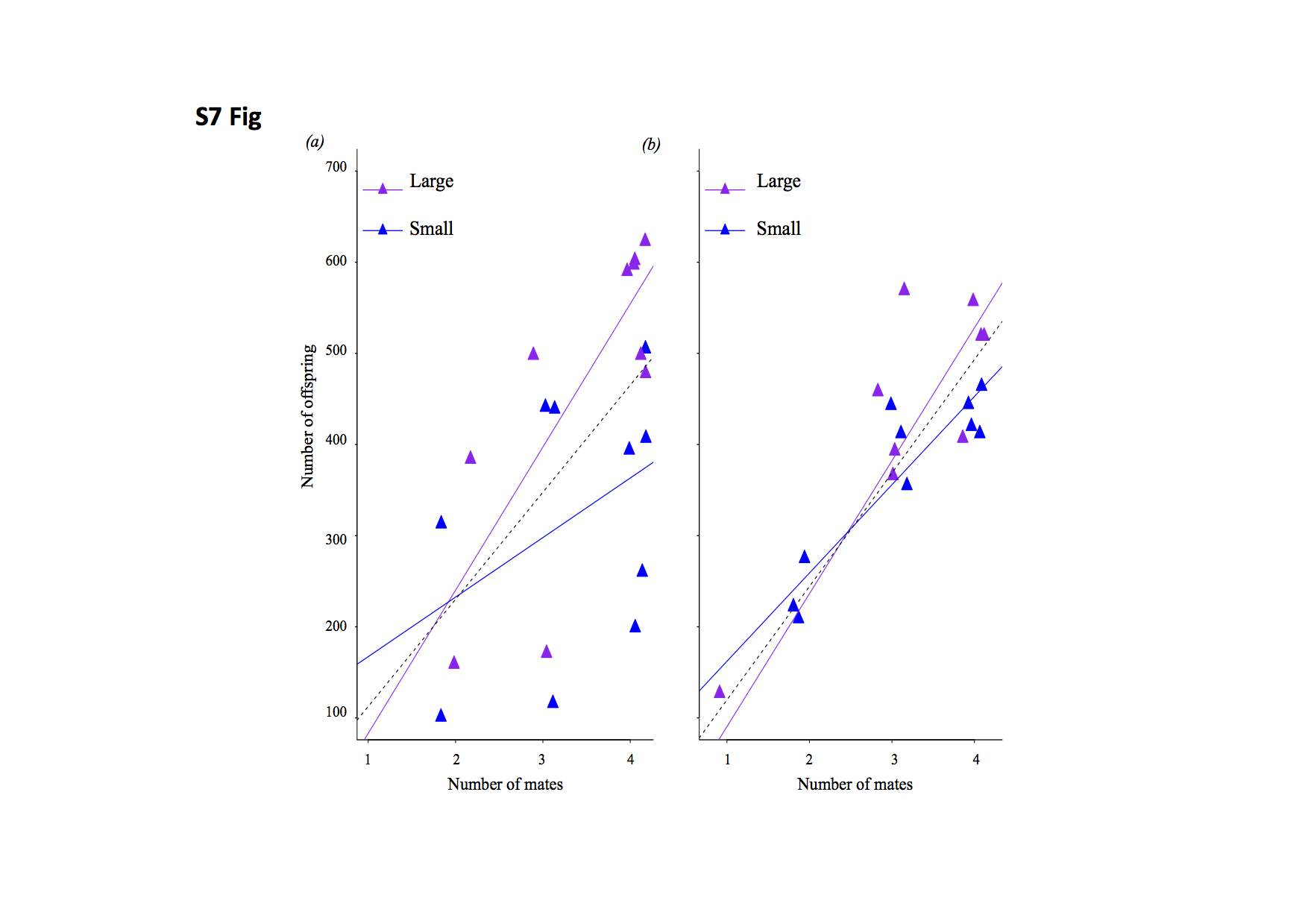

Supplement: S7 Fig — Purple–Large body size; Blue–Small body size; Dashed line–Univariate Bateman gradient. (a) The Male Experiment (b) The Female-Male Experiment; Small (blue)–Large (purple). (TIFF) [file pone.0154468.s007.tiff]
