# Supplementary material for: Developmental Environment Effects on Sexual Selection in Male and Female Drosophila melanogaster
Source: PLoS One. 2016 May 11;11(5):e0154468. doi: 10.1371/journal.pone.0154468 (PMC4864243; doi:10.1371/journal.pone.0154468)
Supplement: S1 Text — Analyses of the effects of colour and changes of sex ratio due to female deaths. (DOCX) [file pone.0154468.s011.docx]

**Supplementary information of the manuscript titled: “Developmental environment effects on sexual selection in male and female *Drosophila melanogaster”***

Authors: Juliano Morimoto^*^, Tommaso Pizzari, Stuart Wigby

Authors affiliation: Department of Zoology, Edward Grey Institute, University of Oxford, South Parks Road, Oxford OX1 3PS, United Kingdom

*To whom correspondence should be addressed.

E-mail: [juliano.morimotoborges@zoo.ox.ac.uk](mailto:juliano.morimotoborges@zoo.ox.ac.uk)

**Supplementary Methods**

*Univariate Bateman gradient* **–** Here, we measured male Bateman gradients using a univariate model. Traditionally, the Bateman gradient *β* was measured as the slope of a linear regression of standardised offspring number on standardised number of mates for both sexes (univariate Bateman gradient; (Arnold and Duvall 1994), as thus:

$$T(M) = (\beta*M)+\varepsilon$$

where $\varepsilon$ is an error term of zero mean. However, the univariate approach tends to overestimate the Bateman gradient, and therefore a multivariate model is required in order to investigate the relative contributions of pre- and prost-copulatory sexual selection for male reproductive success (Collet et al. 2012; Collet et al. 2014) (see main text). Nonetheless, we present the results of the univariate analyses of our data to be consistent with previous literature.

*Commonality analysis (CA)* – CA was performed as suggested by (Ray‐Mukherjee et al. 2014) with the package ‘yhat’ in R software version 3.0.2 (R Development Core Team 2010).

*Sex ratio effects cause by deaths of experimental females (DF) -* Some focal females (but not males) died. To investigate potential effects of these deaths on our results, we pooled the data of our three experiments and created a binary variable called *DF* (dead females)*,* which contained information on the occurrence (value = 1) or not (value = 0) of a female death in the social environment of a particular individual. A significant effect of *DF* means that deaths of experimental females increased or decreased reproduction of the remaining individuals in their social group. We performed GLMs (quasipoisson error distribution to account for overdispersion) to evaluate whether *DF* had a significant effect on the number of mates, mating frequency and number of offspring of both females and focal males (see Table 2 of S1 Text below). Because *DF* had a significant effect on the estimates of the number of mates and offspring of focal males, we included *DF* in all focal male models of reproduction and sexual selection (see Main Text).

**Supplementary results**

*Paint marking –* Colour had no overall effect on the number of offspring and number of mates of either females or focal males (Table 1 of S1 Text).

**Table 1 of S1 Text – The effects of paint marking on male and female reproduction.**

| **Sex** | **Mating frequency** | | **Number of mates** | | **Number of offspring** | |
| --- | --- | --- | --- | --- | --- | --- |
|  | *F-value* | *p-value* | *F-value* | *p-value* | *F-value* | *p-value* |
| *Female* | *0.992* | *0.396* | *0.649* | *0.584* | *1.720* | *0.162* |
| *Focal male* | *1.785* | *0.184* | *2.296* | *0.134* | *3.416* | *0.067* |

*Univariate Male Bateman gradients:*

Standardised univariate male Bateman gradients (Bateman 1948; Arnold and Duvall 1994) were positive, and mostly significant or close to significant across the experiments, with three exceptions: the Bateman gradient for small males with large females in the Male Experiment (HomS^M^), and the homogenous environment with large focal males competing with large male competitors in both the Female and Female-Male Experiments (i.e. HomL^F^ and HomL^FM^) (S1 Table).

*Quadratic effects –* We measured the quadratic effect by adding a quadratic term to the linear model of standard female Bateman gradients. The relationship between offspring production and number of mates might be non-linear for females, suggesting that females maximise offspring production at a given number of mates. Here, we show that non-linear (i.e. negative quadratic effect) relationship between mates and offspring of females. We found a significant negative quadratic effect on the female Bateman gradient in the experimental group Het^M^ of the Male Experiment (Estimate [p-value]; M^2^- -0.046 [0.020]). Negative female quadratic effect was also found in female jungle-fowl (Collet et al. 2014) and suggests that offspring production peaks at a certain number of mates, decreasing thereafter. This pattern is consistent with females having an optimum number of mates.

*Sex ratio effects cause by deaths of experimental females:*

**Table 2 of S1 Text – The effects of changes in sex ratio due to the deaths of experimental females (*DF*).**

| **Sex** | **Factor** | **Mating frequency** | | **Number of mates** | | **Number of offspring** | |
| --- | --- | --- | --- | --- | --- | --- | --- |
|  |  | *F-value* | *p-value* | *F-value* | *p-value* | *F-value* | *p-value* |
| *Female* |  | *0.008* | *0.924* | *1.411* | *0.235* | *1.233* | *0.267* |
| *Focal male* |  | *2.263* | *0.135* | *7.907* | ***0.005*** | *8.875* | ***0.003*** |

**Supplementary references**

Arnold, S. J. and D. Duvall. 1994. Animal Mating Systems - a Synthesis Based on Selection Theory. American Naturalist 143:317-348.

Bateman, A. J. 1948. Intra-Sexual Selection in Drosophila-Melanogaster. Heredity (Edinb.) 2:277-277.

Collet, J., D. S. Richardson, K. Worley, and T. Pizzari. 2012. Sexual selection and the differential effect of polyandry. Proceedings of the National Academy of Sciences 109:8641-8645.

Collet, J. M., R. F. Dean, K. Worley, D. S. Richardson, and T. Pizzari. 2014. The measure and significance of Bateman's principles. Proceedings of the Royal Society B-Biological Sciences 281.

R Development Core Team. 2010. R: a language and environment for statistical computing. R. Foundation for Statistical Computing.

Ray‐Mukherjee, J., K. Nimon, S. Mukherjee, D. W. Morris, R. Slotow, and M. Hamer. 2014. Using commonality analysis in multiple regressions: a tool to decompose regression effects in the face of multicollinearity. Methods Ecol. Evol. 5:320-328.
